# Overexpression of a Grapevine Sucrose Transporter (VvSUC27) in Tobacco Improves Plant Growth Rate in the Presence of Sucrose *In vitro*

**DOI:** 10.3389/fpls.2017.01069

**Published:** 2017-06-20

**Authors:** Yumeng Cai, Wenrui Tu, Yunyun Zu, Jing Yan, Zimo Xu, Jiang Lu, Yali Zhang

**Affiliations:** ^1^Beijing Advanced Innovation Center for Food Nutrition and Human Health, College of Food Science and Nutritional Engineering, China Agricultural University Beijing, China; ^2^Center for Viticulture and Enology, School of Agriculture and Biology, Shanghai Jiao Tong University Shanghai, China

**Keywords:** grapevine, VvSUC27, growth, abiotic stresses, sucrose

## Abstract

The import of sugar from source leaves and it further accumulation in grape berries are considerably high during ripening, and this process is mediated via sucrose transporters. In this study, a grape sucrose transporter (SUT) gene, *VvSUC27*, located at the plasma membrane, was transferred to tobacco (*Nicotiana tabacum*). The transformants were more sensitive to sucrose and showed more rapid development, especially roots, when cultured on MS agar medium containing sucrose, considering that the shoot/root dry weight ratio was only half that of the control. Moreover, all transformed plants exhibited light-colored leaves throughout their development, which indicated chlorosis and an associated reduction in photosynthesis. The total sugar content in the roots and stems of transformants was higher than that in control plants. No significant difference was observed in the leaves between the transformants and control plants. The levels of growth-promoting hormones were increased, and those of stress-mediating hormones were reduced in transgenic tobacco plants. The qRT-PCR analysis revealed that the expression of *VvSUC27* was 1,000 times higher than that of the autologous tobacco sucrose transporter, which suggested that the markedly increased growth rate of transformants was because of the heterogeneously expressed gene. The transgenic tobacco plants showed resistance to abiotic stresses. Strikingly, the overexpression of *VvSUC27* leaded to the up regulation of most reactive oxygen species scavengers and abscisic acid-related genes that might enable transgenic plants to overcome abiotic stress. Taken together, these results revealed an important role of VvSUC27 in plant growth and response to abiotic stresses, especially in the presence of sucrose *in vitro*.

## Introduction

Plants produce carbohydrate metabolites via photosynthesis to support their development. Fixed carbon is subsequently distributed to many tissues, e.g., developing leaves, flowers, fruits, and roots, which cannot reduce carbon alone. In most plants, sucrose is the predominant form of carbon transported through the vascular system to the sink organs. Therefore, sucrose transporters have a pivotal role in the translocation of sucrose.

Since the first sucrose transporter (SUT) was isolated from spinach (Riesmeier et al., 1992), many clones belonging to this gene family have been isolated from various species and plant organs (Derrer et al., 2013). Some of these clones have been shown to be functional SUTs by expressing them in *Xenopus* oocytes or yeast (Deol et al., 2013; Oner-Sieben et al., 2015; Zanon et al., 2015), but their ability to regulate plant metabolism remains unclear.

One method used to elucidate the function of sugar transporters and their physiological roles in plants is to generate plants that have modified transporters (by sense overexpression, antisense inhibition, or gene disruption). Transgenic plants expressing antisense SUTs in potato (Kuhn et al., 1996; Lemoine et al., 1996; Gabriel-Neumann et al., 2011), tobacco (Burkle et al., 1998), or tomato (Bitterlich et al., 2014) have been characterized for their accumulation of leaf carbohydrates and impaired root development. Moreover, reduced tuber yield in potato lines, delayed or impaired flowering in tobacco lines, and increased mycorrhizal colonization in tomato lines have been observed. These results suggest that the antisense expression of SUTs in plants severely reduces plant growth, and that SUTs in the phloem are essential for carbohydrate partitioning, at least in tobacco, potato, and tomato (members of Solanaceae). Further, studies have investigated the effects of sucrose transporter overexpression. The overexpression of the *Hordeum vulgare* SUT (HvSUT1) increased grain protein content and deregulated the metabolic status of wheat (*Triticum aestivum*) grains (Weichert et al., 2010). Potato plants transformed with a sense spinach SUT (SoSUT1) showed reduced sucrose levels in leaves and varied tuber morphology (Leggewie et al., 2003). Transgenic potato plants with constitutive overexpression of *SUT1* exhibited increased mycorrhization (Gabriel-Neumann et al., 2011). The overexpression of a potato *SUT* (*StSUT1*) in pea cotyledon storage parenchyma cells enhanced the sucrose uptake capacity of cotyledons and their growth rates (Rosche et al., 2002). However, this enhancement was achieved in a single organ, rather than in the entire plant.

Three putative SUT (*VvSUC11, VvSUC12*, and *VvSUC27*) cDNAs have been identified in grape (*Vitis*) berry (Davies et al., 1999). Their abilities to transport sucrose have been shown in a heterologous yeast expression system; considering the value of *K*_*m*_ to sucrose, VvSUC11 and VvSUC12 are intermediate-affinity SUTs (*K*_*m*_ of 0.88 and 1.36 mM, respectively; Ageorges et al., 2000; Manning et al., 2001), and VvSUC27 is a low-affinity/high-capacity (LAHC) sucrose transporter (*K*_*m*_ of 8.0–10.5 mM; Zhang et al., 2008). Northern blot analysis was used to determine the expression of these three SUTs in different tissues; *VvSUC11* and *VvSUC12* were found to be expressed in numerous tissues, except in roots and tendrils. However, *VvSUC27* was highly expressed in the roots, petioles, stems, young leaves, and tendrils and was expressed at lower levels in berries and old leaves. Thus, *VvSUC27* is a sink-specific gene, which is responsible for phloem loading and sugar retrieval during long-distance transport and is closely related to members of the SUT1 subfamily (Davies et al., 1999; Afoufa-Bastien et al., 2010). However, during berry development, the transcript abundance of *VvSUC27* was lower than that of the other two SUTs assessed (*VvSUC11* and *VvSUC12*); the decreased transcript abundance was exhibited especially after veraison (Davies et al., 1999; Deluc et al., 2007; Afoufa-Bastien et al., 2010; Pastenes et al., 2014; Xu et al., 2015). Notably, a small portion of plasmodesmata was apparently blocked in the ripening stage, and the phloem strands were blocked during the late stage (Zhang et al., 2006). These phenomena might be associated with a shift in phloem unloading from the symplasmic to apoplasmic pathway during berry development. However, studies focusing on the function of VvSUC27 are not systematic, and the effect of increased VvSUC27 activity on plant properties has not been determined. Previously, we isolated and characterized VvSUC27 in a heterologous yeast strain to characterize its function as a LAHC SUT; its sucrose uptake activity was activated in transformed yeast by monosaccharides and inhibited by maltose and diethyl pyrocarbonate (Zhang et al., 2008). In this study, in order to further investigate the function and effect of VvSUC27 on a genetically modified plant phenotype and the related mechanisms, we determined the gene expression pattern and subcellular localization of VvSUC27 and generated transformants. Our results indicated that *VvSUC27* is a candidate gene to improve plant growth and abiotic stress tolerance *in vitro*, especially in the presence of sucrose.

## Materials and methods

### Plant material and culture condition

Grapevine berry of *V. riparia* DVIT1848, Frontenac (*V. riparia* × *Landot* 4511), *V. amurensis* Ruper. “Zuoshan-1,” *V. amurensis* Ruper. “Zuoshan-2,” and *Vitis vinifera* L. “Cabernet Sauvignon” were collected from Shangzhuang (Beijing, China). *V. vinifera* L. “Cabernet Sauvignon” was collected about 20 weeks post-flowering.

The culture medium was solid Murashige and Skoog (MS) medium (not containing sucrose). The plant material used was grown on agar MS with or without 30 g·L^−1^ sucrose. All plants were grown *in vitro* at a constant temperature of 25°C with a 16/8-h day/night regime (150 μmoL·m^−2^·s^−1^).

### Total RNA isolation

Total RNA was isolated as previously described with some modifications (Zhang et al., 2008). Fresh tissue was cut from live plants, frozen in liquid nitrogen, and stored at −80°C. RNA was isolated by grinding 0.2 g of tissue to a fine powder with a mortar and pestle pre-chilled with liquid nitrogen. The powder was transferred to a warm cuvette containing 0.7 mL of extraction buffer (2% CTAB, 2% PVP, 25 mM EDTA, 100 mM Tris–HCl, pH 8.0, 2.0 M NaCl) and 14 μL of β-mercaptoethanol and ground for 30–45 s to thoroughly mix the material. The homogenate was incubated in a 65°C water bath for 10 min, and then extracted with an equal volume of chloroform/isoamyl alcohol (24:1), shaked. The two layers were separated by centrifugation at 13,000 × g for 15 min. The aqueous phase was collected and extracted with an equal volume of chloroform/isoamyl alcohol (24:1) again, centrifuged at 13,000 × g for 15 min. The aqueous phase was collected and the total RNA was precipitated with a 1/4 volume of 10 M LiCl at −20°C for 6 h. The nucleic acids were collected by centrifugation at 13,000 × g for 30 min at 4°C and then rinsed with 70% ethanol once. After the pellet was dried, it was resuspended in 100 μL DEPC water.

### Isolation of *VvSUC27* cDNA

Fresh grape tissue was cut from live plants, frozen in liquid nitrogen, and stored at −80°C for total RNA preparation. Total RNA was isolated from *V. vinifera* L. “Cabernet Sauvignon” as described. The FastQuant RT Kit (with gDNase; Tiangen, China) was used for the synthesis of cDNA as per manufacturer's instructions. Oligo dT was used as the primer for cDNA synthesis. The cDNA was designed with respect to the cDNA encoding VvSUC27 (AF021810; Davies et al., 1999). The oligonucleotide probes used were as follows:

5′-ATGGAGTTAGCCAAGCCTTCTTC-3′
5′-TTAAGACGACGGCTGAGTCCTC-3′

These probes were used to generate a clone with a full-length 1,518-bp PCR product, which contained the sequence of the *VvSUC27* gene. The fragment was purified and ligated into a pGEX-T vector (Promega).

### Preparation of transgenic plants

The binary vector pBI121 was used to carry two cassettes between the T-DNA borders (Nowak et al., 2001): (i) the *npt-II* gene conferring kanamycin resistance to the transformants; and (ii) the glucuronidase (*GUS*) gene, whose transcription is under the control of the 35S promoter. In our study, any side-effect due to the *GUS* gene were avoided by replacing it by the *VvSUC27* gene. The *VvSUC27* gene was inserted in sense orientation by using the *Bam*HI and *Sac*I. Plasmid integrity was verified using restriction enzyme analysis. The binary vectors were transferred to *Agrobacterium tumefaciens* LBA 4404, and plants were transformed using the leaf disc transformation procedure (Graybosch et al., 1989). Tobacco (*N. tabacum* “Samsun”) plants were grown on sterilized MS medium at 25°C, with a 16-h photoperiod. Rooted plants from sterile cultures were transferred to pots containing sterilized garden soil and grown under an 11-h photoperiod at 25°C. The plants were allowed to flower and self-fertilize, and the seeds were collected. Homozygotes of T_3_ seedlings were used for further analyses.

### DNA isolation and southern blotting

Genomic DNA was isolated from the supernatant of LiCl RNA precipitation by adding 2 volumes of ethanol, followed by centrifugation at 5,000 × g for 15 min. The DNA pellet was resuspended in water and treated with RNase (1 g·L^−1^) and proteinase K20 (50 mg·L^−1^) for 60 min at 37°C. Following extraction with phenol–chloroform–isoamyl alcohol (25:24:1, by volume), the DNA was precipitated by the addition of isopropanol. The pellet was washed with 70% ethanol and dissolved in water. The DNA (7–10 μg) was digested with *BamH*I and *Sac*I for 16 h. The DNA fragments were separated on a 0.8% agarose gel overnight at 30 V and transferred onto Hybond N^+^ membranes (Amersham Life Science). Southern blot was performed using the DIG High Prime Labeling and Detection Starter Kit (Roche) as described by the manufacturer.

### Uptake activity

Uptake experiments were performed using 8-week-old tobacco (*N. tabacum* “Samsun”) plants. The previously reported method was used for reference (Leterrier et al., 2003); however, because a different transporter was investigated in this study, sucrose was used as the substrate. The incubation medium contained 1 mM sucrose and 0.1 MBq mL^−1^sucrose-[^14^C] (Amersham Pharmacia Biotech, Buckinghamshire, UK). The sucrose concentration was determined using the *K*_*m*_, which is usually determined for plant SUTs in the plasma membrane vesicles (0.2–1 mM; Lemoine, 2000), and is expected to saturate the transporter without any strong contribution by a diffusional component.

### Localization study

The *VvSUC27* for subcellular localization was cloned by PCR amplifying the open reading frame (ORF) of its cDNA. The primers used are shown in Table S1. The pUC19-CaMV 35S-*VvSUC27*-GFP plasmid was used for transient expression in *Arabidopsis* protoplasts. Protoplasts were isolated from the leaves of 3- to 4-week-old plants of *Arabidopsis* (ecotype Columbia), transiently transformed using polyethylene glycol, and incubated at 23°C for 16 h (Ueda et al., 2001). We used the fusion plasmid pBI121-CaMV 35S-*VvSUC27*-GFP, which was expressed transiently in *Nicotiana benthamiana* via agroinfiltration to determine the subcellular localization of VvSUC27 as previously described (Xiang et al., 2016). Green fluorescent protein (GFP) fluorescence was observed using a Nikon C1 Si/TE2000E confocal laser scanning microscope, and the EZ-C1 3.00 software was used for image processing.

### Seedling growth experiments

Seedling growth experiments were performed using solid MS media containing sucrose concentrations ranging from 0 to 6% (g·100 mL^−1^). The seedling growth ratio (%, the number of germinated seeds/the number of whole seeds in each bottle) of the transformed tobacco seeds on media containing different sucrose concentrations was investigated for 9 d. The inhibition rate of each line was calculated as follows:

(1) $$\begin{array}{r} IR=\frac{\text{SRx}-\text{SRo}}{\text{SRo}}\times 100\%, \end{array}$$

Where SR_*x*_ and SR_*o*_ represent the seedling growth ratio on solid MS media containing the corresponding sucrose concentration and that without sucrose, respectively.

### Growth conditions and phenotype analysis

Plants were grown in a greenhouse at a constant temperature of 25°C with a 16/8-h day/night regime (150 μmoL·m^−2^·s^−1^) on MS medium containing 0.7% (w/v) agar (pH 5.8) in the absence and presence of 30 g·L^−1^ exogenous sucrose. Seeds from each transformant and control (CK) were planted on MS medium supplemented with or without 30 g·L^−1^ exogenous sucrose in 200 mL culture vessels with 12 cm height (50 mL medium in each vessel). Thirty-day-old seedlings were used to determine the stem diameter, leaf number, leaf area, and root number. Stem length was recorded every week for 7 weeks. For stress resistance assay, seeds were planted on the medium supplemented with 0.15 M NaCl or 0.2 M mannitol.

### Dry weight determination

Four-to-six transformed tobacco plants for each independent clone were cultured *in vitro* for 6 weeks. Roots, leaves, and stems of each plant were cut and weighed separately after 24-h incubation at 80°C.

### Sugar measurements

Fresh leaves, stems, and roots were separated from the plants, placed at 105°C for 15 min, and then dried at 75°C overnight until a constant weight was reached. The dry tissue was ground using a mortar and pestle. The dry powder (25 mg) was dissolved in 1.5 mL 80% ethanol and distilled at 80°C for 30 min, modified anthrone-sulfuric acid method for further determination. Measurements were performed as previously described (Somani et al., 1987; Zhang et al., 2016).

### Determination of endogenous phytohormone content

Seven-week-old seedlings were collected following planting on MS medium with or without 30 g·L^−1^ exogenous sucrose supplementation. Endogenous methyl jasmonic acid (MeJA), salicylic acid (SA), indole-3-acetic acid (IAA), abscisic acid (ABA), and gibberellic acid (GA_3_) were extracted from the transformed (Lines 9, 15, and 16) and CK as described previously (Pan et al., 2010) and measured using a high-performance liquid chromatography-mass chromatography system (HPLC-MS) (Varian, USA) by the Beijing Center for Physical and Chemical Analysis.

### Stress growth conditions and phenotype analysis

For the stress resistance assay, seeds were planted on 0.7% (w/v) agar MS medium (pH 5.8) supplemented with 0.15 M NaCl or 0.2 M mannitol and no exogenous sucrose or 30 g·L^−1^ exogenous sucrose under a 16/8-h day/night regime (150 μmoL m^−2^ s^−1^). For the light experiment, seedling growth in MS media containing 30 g·L^−1^ sucrose was investigated for 30 d, after which they were placed under low light (75 μmoL m^−2^ s^−1^) or no light conditions for further 30 d. All plants were grown in a greenhouse at a constant temperature of 25°C. Stem diameter, stem length, leaf number, leaf area, and root number were measured.

### Quantitative real-time PCR

RNA was extracted from four kinds of frozen grapes or plant leaves, stems, and roots of CK and the three overexpression lines; the samples were treated with DNase as described above. Quantitative real-time PCR (qRT-PCR) was performed using SYBR Premix Ex Taq (TAINGEN, China) by using appropriate gene-specific primers (Table S1). The threshold cycle for each PCR with different concentrations of cDNA was determined and compared against a standard DNA (*EF1*α) that was also analyzed simultaneously (Wang et al., 2014). A ratio of the concentration of gene-specific mRNAs in the sample was subsequently calculated.

### Statistical analyses

One-way analysis of variance (ANOVA) and a subsequent Tukey's test were performed to analyze data by using SPSS16.0 (SPSS Corp., Chicago, IL, USA). A *P* < 0.05 was considered statistically significant. Relationships between *VvSUC27* expression and sugar accumulation were explored using Pearson's correlation co-efficient. Pearson's *r* was always between −1 and +1, where −1 refers to a perfect negative relationship; +1, to a perfect positive relationship; and 0, to the absence of a relationship. All experiments were repeated three times.

## Results

### *VvSUC27* expression is negatively correlated with sugar accumulation

**Figure 1:**
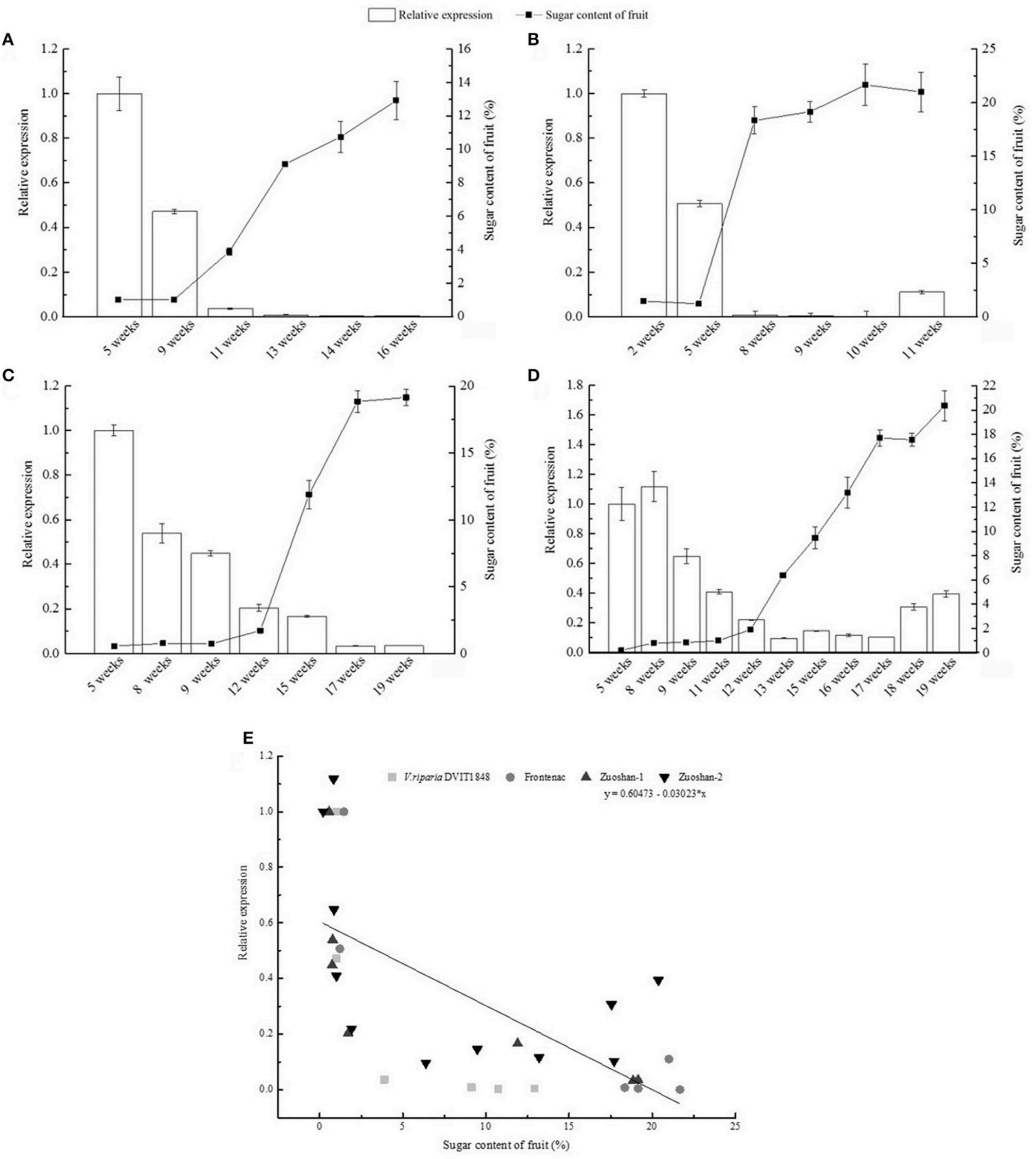
Relationship between *VvSUC27* gene expression and sugar content in grapevine berry of *V. riparia* DVIT1848 **(A)**, Frontenac (*V. riparia* × *Landot* 4511) **(B)**, *V. amurensis* Ruper. Zuoshan-1 **(C)**, *V. amurensis* Ruper. Zuoshan-2 **(D)** during development after flowering. Data are expressed as the mean ± *SD*. from six independent experiments. Pearson correlation coefficient was calculated to determine the negative correlation between *VvSUC27* gene expression and sugar content (*r*^2^ = 0.43, Pearson's *r* = −0.67, *P* < 0.001) **(E)**.

The relationship between *VvSUC27* gene expression and sugar accumulation was investigated by isolating total RNA and sugars from grape berry tissue during berry ripening. Sugar content was relatively low in the beginning and markedly increased after veraison (Figures 1A–D). In contrast, a relatively high level of *VvSUC27* expression was noted in berries before veraison. However, after veraison, its level remarkably decreased. The data obtained from four different varieties of grape suggested a negative correlation between *VvSUC27* gene expression and sugar accumulation. This finding was confirmed by conducting a correlation analysis (Figure 1E). A strong negative correlation was noted between *VvSUC27* gene expression and sugar accumulation, suggesting that VvSUC27 was probably responsible for sugar retrieval during long-distance transport. Therefore, we selected this gene for further study.

### Cloning of VvSUC27 into an expression vector and transgenic tobacco production

Total RNA was isolated from grape berry tissue, VvSUC27 (AF021810) cDNA was amplified using RT-PCR, and a 1518-bp fragment was cloned into the pGEM-T easy vector. After the vector was double-digested with *Bam*HI/*Sac*I, the coding region was ligated to the binary vector pBI121 in the sense orientation (Figure S1A).

The construct and blank plasmid were used to transform tobacco leaf discs by using the *Agrobacterium*-mediated transformation method. Fifteen lines that showed positive results in Southern blot analysis (Figure S1B) were cultured on antibiotic-selection medium until new roots and shoots emerged; they were transferred to the greenhouse for growth until seeds were produced. The primary tobacco transformants (T_0_) grown from the transformed leaf discs expressing *VvSUC27* cDNA were selfed to generate the T_1_ generation. Phenotypic differences between the transformants and CK plants were not visible in the field. The homozygotes of T_3_ seedlings were chosen for further analysis.

### VvSUC27 as a functional SUT is located at the plasma membrane

**Figure 2:**
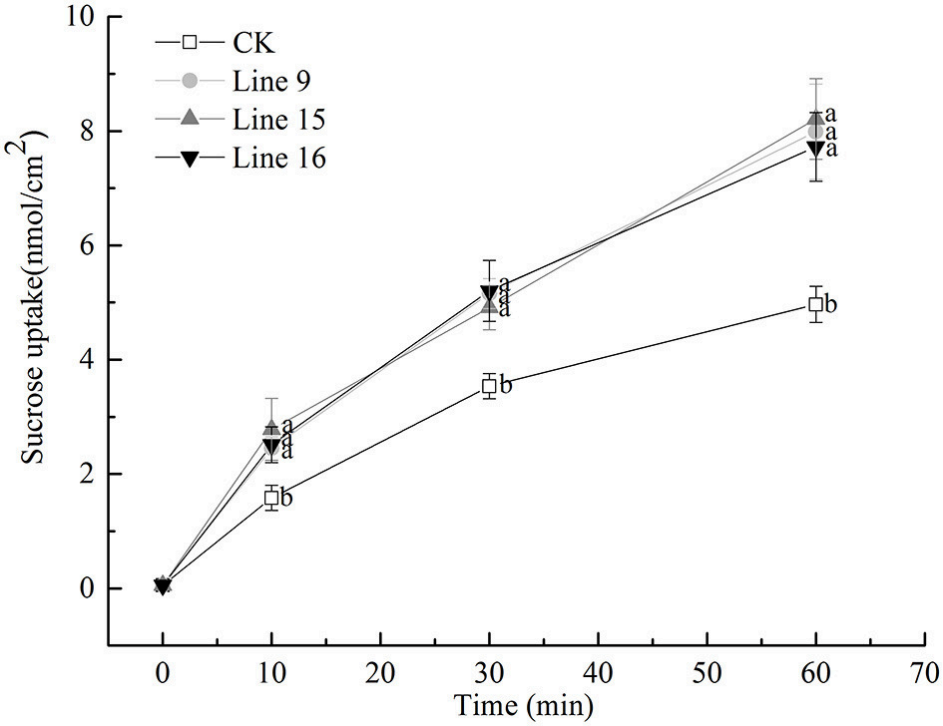
Uptake rate of sucrose-[^14^C] (1 mM) by leaf discs of VvSUC27-transformed tobacco plants. Data are expressed as the mean ± *SD* of determinations from three independent samples. Analysis for each sample was repeated three times, and similar results were obtained. Different letters indicate significant differences (*P* < 0.05) differences between transformants (Lines 9, 15, and 16) and CK, as determined by one-way analysis of variance followed by Tukey's test using SPSS statistical software.

**Figure 3:**
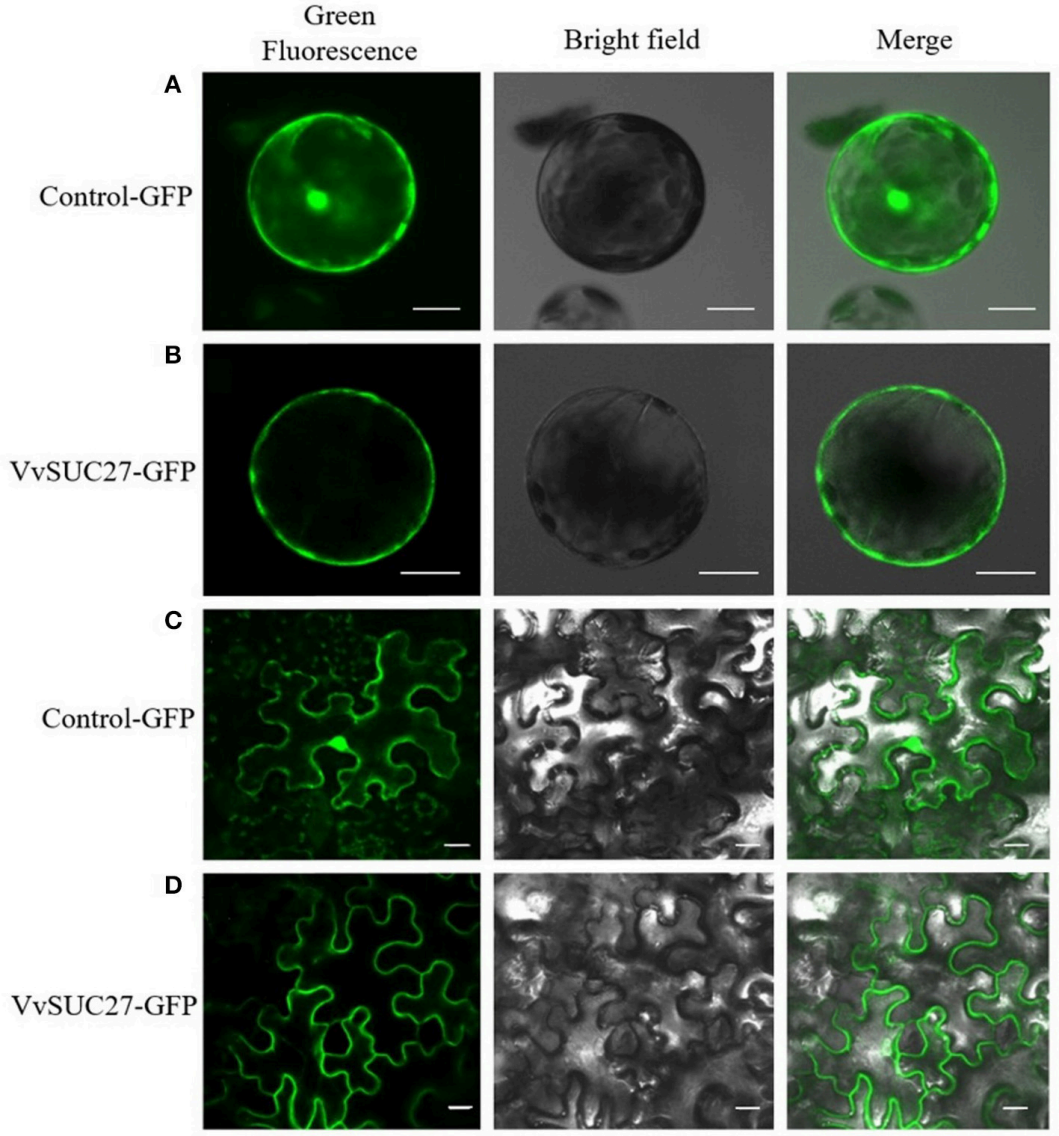
Subcellular localization of VvSUC27 by transient expression of GFP fusion proteins in *Arabidopsis* protoplasts and *Nicotiana benthamiana*. Transient expression of GFP (as control) and VvSUC27-GFP under the control of the 35S promoter *Arabidopsis* protoplasts **(A,B)** and *N. benthamiana* **(C,D)**. Localization of GFP to the plasma membrane, cytoplasm, and nucleus in *Arabidopsis* protoplasts **(A)** and *N. benthamiana* **(C)**. Plasma membrane localization of VvSUC27 in *Arabidopsis* protoplasts **(B)** and *N. benthamiana* **(D)**. Bars = 10 μm.

The function of VvSUC27 as a SUT was confirmed again by monitoring the uptake of sucrose-[^14^C] by the leaf tissues; all the transformants showed a significantly high capacity for sucrose uptake into source leaves (Figure 2), which indicated that VvSUC27 was located at the plasma membrane. The location of VvSUC27 in the plasma membrane was further confirmed by subcellular localization. The C-terminal end of VvSUC27 was fused to GFP and expressed under the control of the CaMV 35S promoter [pUC19-CaMV 35S-*VvSUC27*-GFP or pBI121-CaMV 35S-*VvSUC27*-GFP] to determine its subcellular localization by monitoring GFP fluorescence in *Arabidopsis* protoplasts and *N. benthamiana* (Figure 3). In *Arabidopsis* protoplasts and *N. benthamiana* transformed with control vector [pUC19-GFP and pBI121-GFP, respectively], green fluorescence was distributed in the cytoplasm, nucleus, and plasma membrane of the transgenic cells (Figures 3A,C). However, green fluorescence was exclusively detected in the plasma membrane of cells transformed with the fusion plasmid (Figures 3B,D). These results indicated that VvSUC27 was localized to the plasma membrane.

### *VvSUC27* overexpression lines show a stronger developmental phenotype when grown on sucrose medium

**Figure 4:**
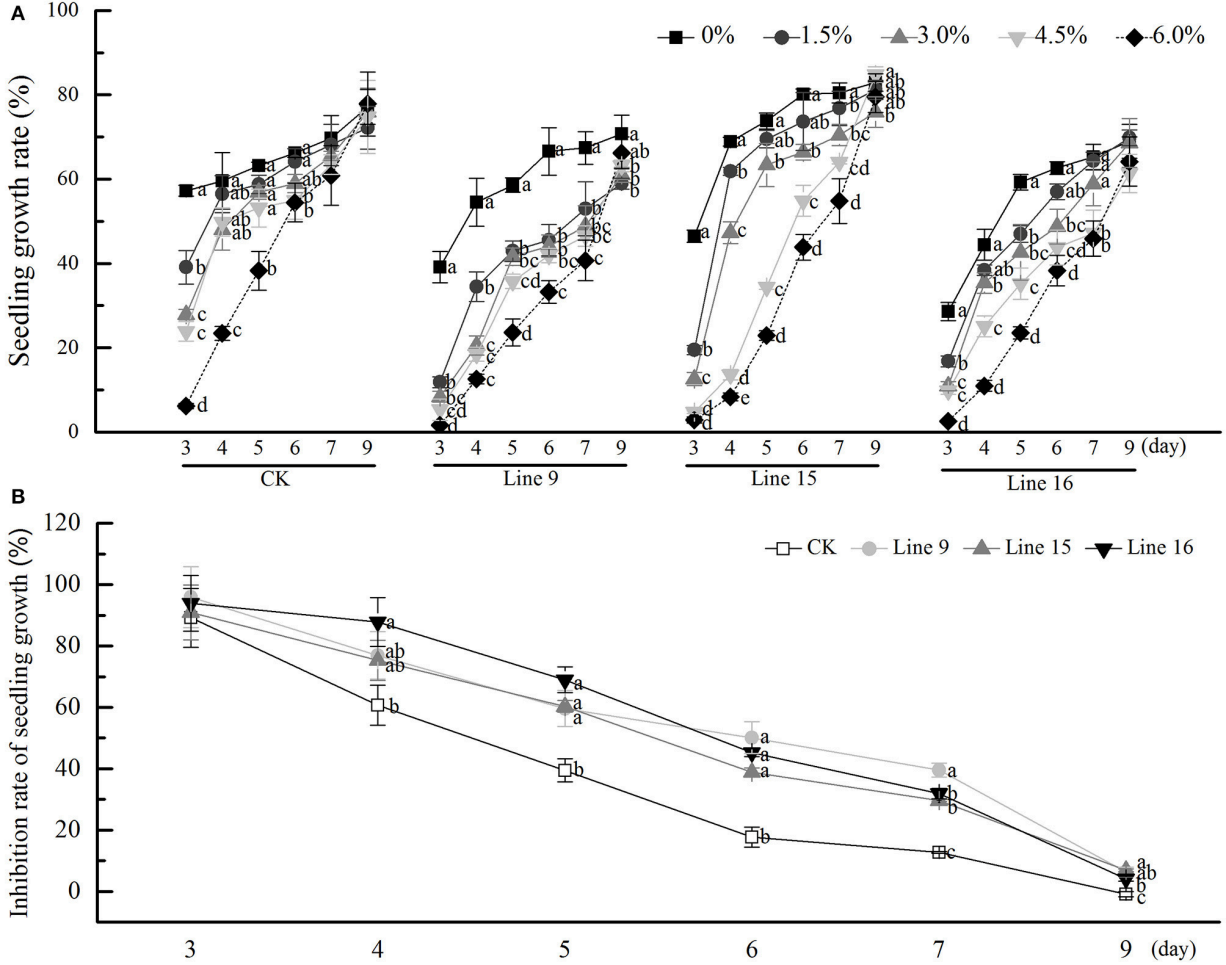
Effects of sucrose concentration on the seedling growth of transgenic tobacco. Seedling growth rate of transgenic tobacco in Lines 9, 15, and 16 under different sucrose concentrations. **(A)** Rate of Seedling growth inhibition. Transgenic and control seeds were compared on MS medium containing 60 g·L^−1^ sucrose **(B)**. Data are expressed as the mean ± *SD* from six independent experiments. Different letters indicate significant differences (*P* < 0.05) differences between transformants (Lines 9, 15, and 16) and CK, as determined by one-way analysis of variance followed by Tukey's test using SPSS statistical software.

**Figure 5:**
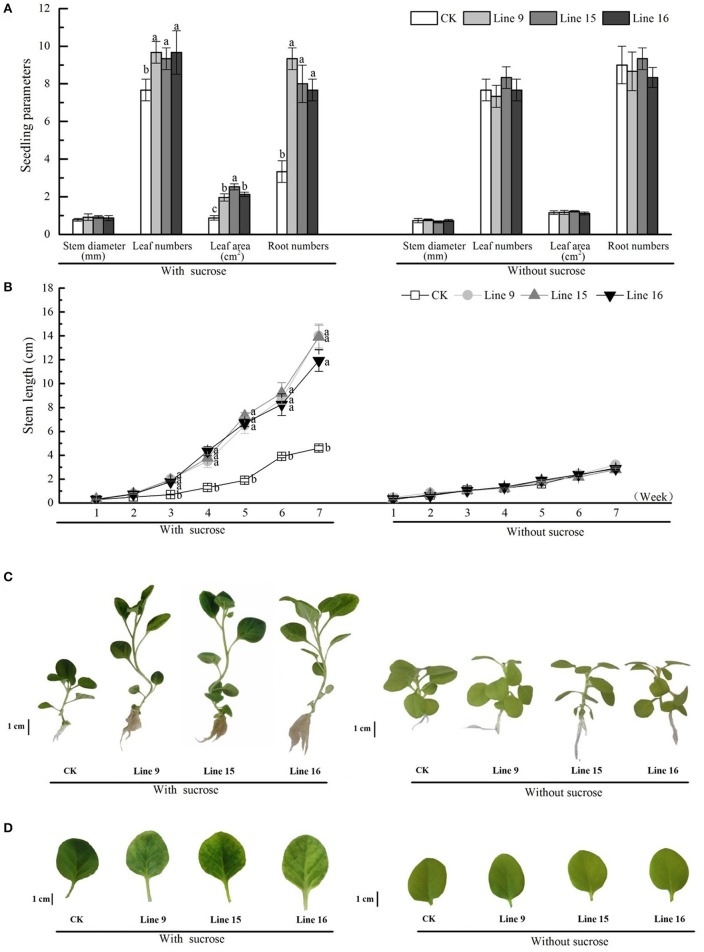
Phenotypes of VvSUC27-transformed tobacco plants. The plants were grown *in vitro* on MS medium containing 30 g·L^−1^ sucrose or without sucrose. Thirty-day-old seedlings were used to investigate stem diameter, leaf number, leaf area, and root number **(A)**. Time-course of stem growth in transgenic tobacco plants and CK grown *in vitro*. Stem length (between the root/shoot junction stem to the base of the growing apex) was measured over 7 weeks **(B)**. Data are expressed as the mean ± *SD* from six independent experiments. Different letters indicate significant differences (*P* < 0.05) differences between transformants (Lines 9, 15, and 16) and CK, as determined by one-way analysis of variance followed by Tukey's test using SPSS statistical software. Phenotypic development in 7-week-old seedlings of transformants compared with that in 7-week-old seedlings of CK **(C)**. Leaves were cut from 7-week-old transformed plants and CK **(D)**.

We investigated the inhibitory effects of exogenous sucrose on the seedling growth of transgenic tobacco and found that higher sucrose concentrations resulted in a lower seedling growth rate, till day 9, relieved inhibition (Figure 4A). The inhibition rate of each line was compared with that of CK at a sucrose concentration of 6% (Figure 4B), and the transformants showed greater inhibition than CK. Then the seeds of transformants were sterilized and germinated on MS medium containing 30 g·L^−1^ sucrose. Compared with CK, transformants had root systems that were noticeably more developed, had taller and thicker stem, and included more and larger leaves (Figures 5A,B). Among the 15 transformant lines, only two lines (Lines 5 and 8) showed no visible phenotypic distinctions compared to CK (data not shown). The visible phenotype of transformants was shown not only in the remarkable improvement in performance, but also in terms of chlorosis observed in mature leaves (Figures 5C,D). This phenotype was observed after the third week. Almost all leaves (except cotyledons) of transgenic tobacco were yellow, and this phenotype was observed in all 13 transgenic lines and throughout their development *in vitro* (data not shown). The yellow region was mainly noted at the tips of mature leaves, indicating a reduction in photosynthesis. However, transformants grown on MS medium without sucrose, as in the control condition, were visually indistinct from the control (Figure 5). A little difference in morphology was noted between their roots, stems, and leaves (Figures 5A,B), and the leaves were of the same color (Figures 5C,D).

**Figure 6:**
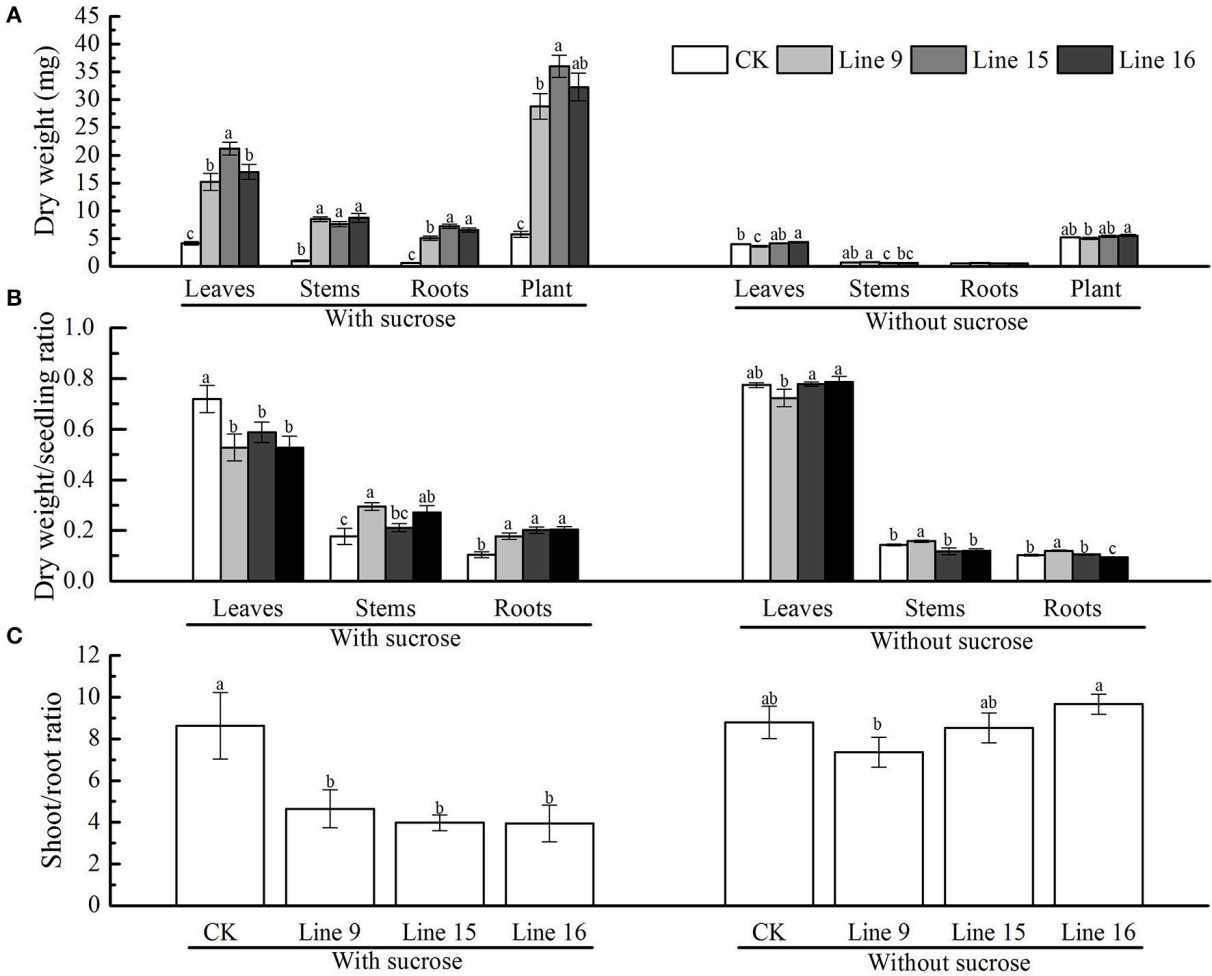
Dry weight and shoot/root ratio of VvSUC27-transformed tobacco plants. The plants were grown *in vitro* for 7 weeks after being plated on modified MS medium containing 30 g·L^−1^ sucrose or without sucrose. The dry weight of the total plant and different plant parts (leaves, stems, roots) **(A)**, the dry weight/seedling ratio **(B)**, and the shoot/root ratio **(C)** of transgenic tobacco plants (Lines 9, 15, and 16) and CK were measured. Data are expressed as the mean ± *SD* from six independent experiments. Different letters indicate significant differences (*P* < 0.05) differences between transformants (Lines 9, 15, and 16) and CK, as determined by one-way analysis of variance followed by Tukey's test using SPSS statistical software.

The dry weight of the different parts of transformants grown on MS medium containing 30 g·L^−1^ sucrose was determined at 6 weeks after germination. At least six plants were obtained and separated into leaves, stems, and roots. The mean values for each line are shown in Figure 6A. Although, the significant differences existed between certain transgenic lines, they were all significantly different from CK, the weight of roots, stems, and leaves gained significantly, which resulted in the total plant dry weight markedly increased. The dry weight/seedling ratio of root and stem were significantly gained, while the ratio markedly decreased in leaves (Figure 6B). The shoot/root ratio indicated that transformants had a relatively large and strong root system (Figure 6C). Similar to that in CK, the dry weight of different transgenic plant parts and the shoot/root ratio of the plants grown on MS medium without sucrose were visually indistinct (Figure 6). The mean dry weight values for leaves, stems, and roots in each plant (all three transformants and CK) were almost 4, 0.7, 0.55, and 5 mg, respectively (Figure 6A). Differences in the dry weight/seedling and shoot/root ratio for each plant were very small (Figures 6B,C).

### *VvSUC27* overexpression lines have altered photosynthesis, sugar metabolism, and endogenous phytohormone levels when grown on sucrose medium

**Figure 7:**
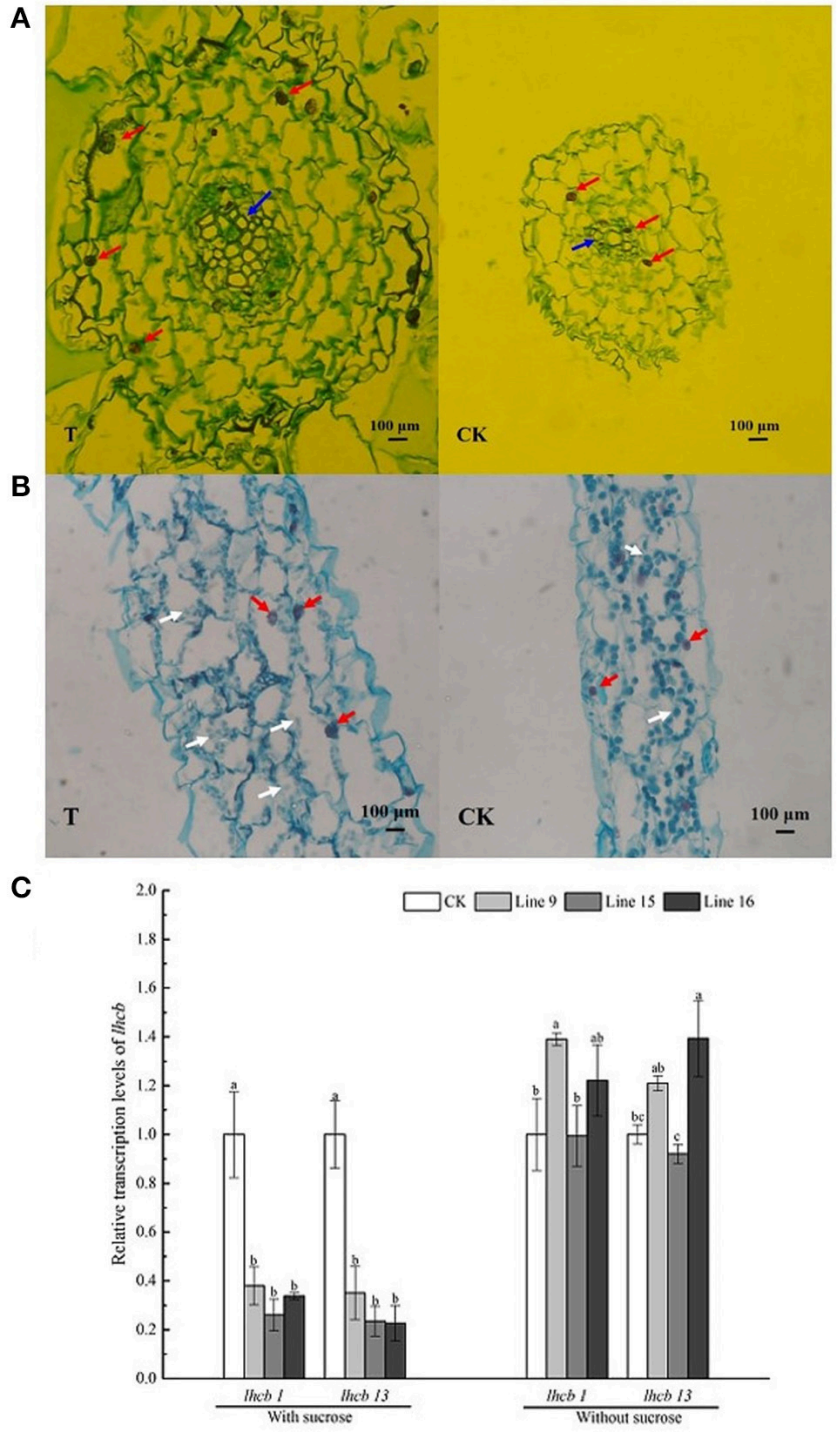
Cross-section of the root and leaf and expression of chlorophyll-related genes in VvSUC27 transformants and CK. Organic slice analysis of root **(A)** and leaf **(B)**. Organic slices were stained by Safranin O/Fast Green staining (SO/FG). Karyons, sieve element, and chloroplasts are indicated by red, blue, and white arrowheads, respectively. Transcript ratios of light harvesting chlorophyll a/b-binding protein (*lhcb*) in transformants (Lines 9, 15, and 16) compared with that in CK **(C)**. The plants were grown *in vitro* for 7 weeks after being plated on MS medium containing 30 g·L^−1^ sucrose. Data are expressed as the mean ± *SD* from six independent experiments. Different letters indicate significant differences (*P* < 0.05) differences between transformants (Lines 9, 15, and 16) and CK, as determined by one-way analysis of variance followed by Tukey's test using SPSS statistical software.

The roots and leaves of VvSUC27 cDNA-transformed tobacco plants showed markedly different phenotypes. Their microstructure was analyzed by performing slice analysis on the root and leaf organs of both transformants and CK (Figure 7).

The roots of transformants had more cell layers, and each cell in the roots had larger volume in cross-section in the transformants than in CK (Figure 7A). Karyons (Figure 7A, indicated by red arrowheads) of the transformed cells were larger. The most distinct section was the sieve element (Figure 7A, indicated by blue arrowheads), which was considerably more developed in the transformants. The leaves of transformants had numerous cells, but the chloroplasts in the leaves were of a smaller size (Figure 7B, indicated by white arrowheads). Furthermore, the expression of the photosynthetic gene *light harvesting chlorophyll a/b binding protein (lhcb*) in the leaves was also investigated (Figure 7C; Kim et al., 2016). RNA isolated from CK was used as a negative control. Expression of the photosynthetic genes *lhcb1* and *lhcb13* were significantly inhibited in the leaves of all transformants. Further, the expression of the photosynthetic gene *lhcb* in transgenic leaves under control conditions (MS medium without sucrose) was unregulated; two lines exhibited higher expression among them only one line of each genes showed significant differences and one line exhibited slightly lower expression than that of CK.

**Figure 8:**
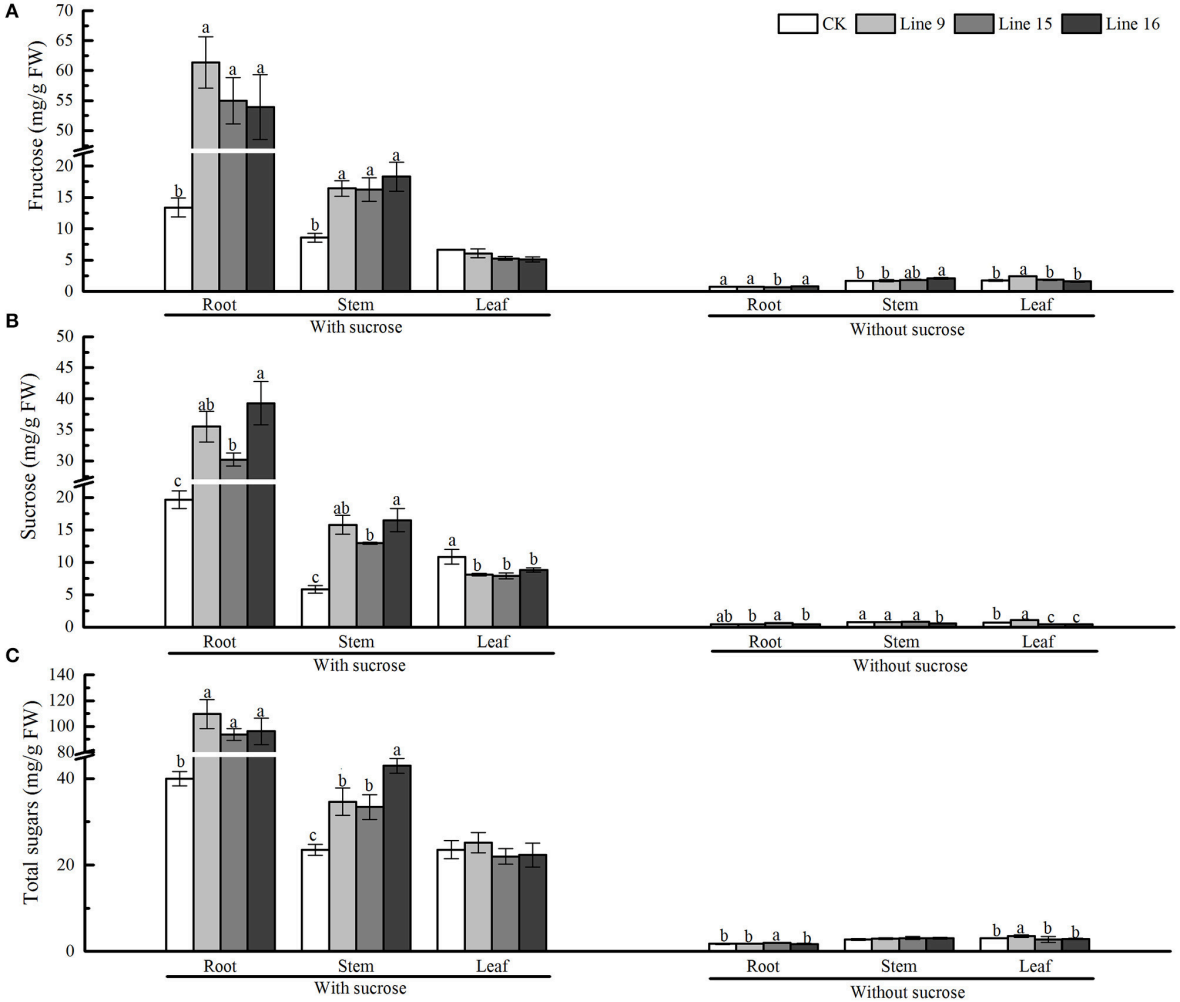
Sugar content of VvSUC27-transformed tobacco plants compared with that of CK. The plants were grown *in vitro* for 7 weeks after being plated on modified MS medium containing 30 g·L^−1^ sucrose or without sucrose. Fructose, sucrose, and total sugar content in the root **(A)**, stem **(B)**, and leaf **(C)** of transformants (Lines 9, 15, and 16) and CK were measured separately. Data are expressed as the mean ± *SD* from six independent experiments. Different letters indicate significant differences (*P* < 0.05) differences between transformants (Lines 9, 15, and 16) and CK, as determined by one-way analysis of variance followed by Tukey's test using SPSS statistical software.

Next, we intended to determine whether the overexpression of VvSUC27 also affected sugar metabolism. Different parts of the transgenic plants and CK (roots, stems, and leaves) were separated, and the carbohydrate contents of each part were measured (Figure 8). On the sucrose medium, the sugar content in the roots of transformants was significantly higher than that in CK. The total sugars in the roots of transformants were 2.7- (Line 9), 2.3- (Line 15), and 2.4-fold (Line 16) higher than those in the roots of CK. The sugar contents in the stems of transformants were also significantly higher than those in CK, however, except for the marked drop in sucrose content, no significant differences were observed in the content for other sugars in the leaves.

**Figure 9:**
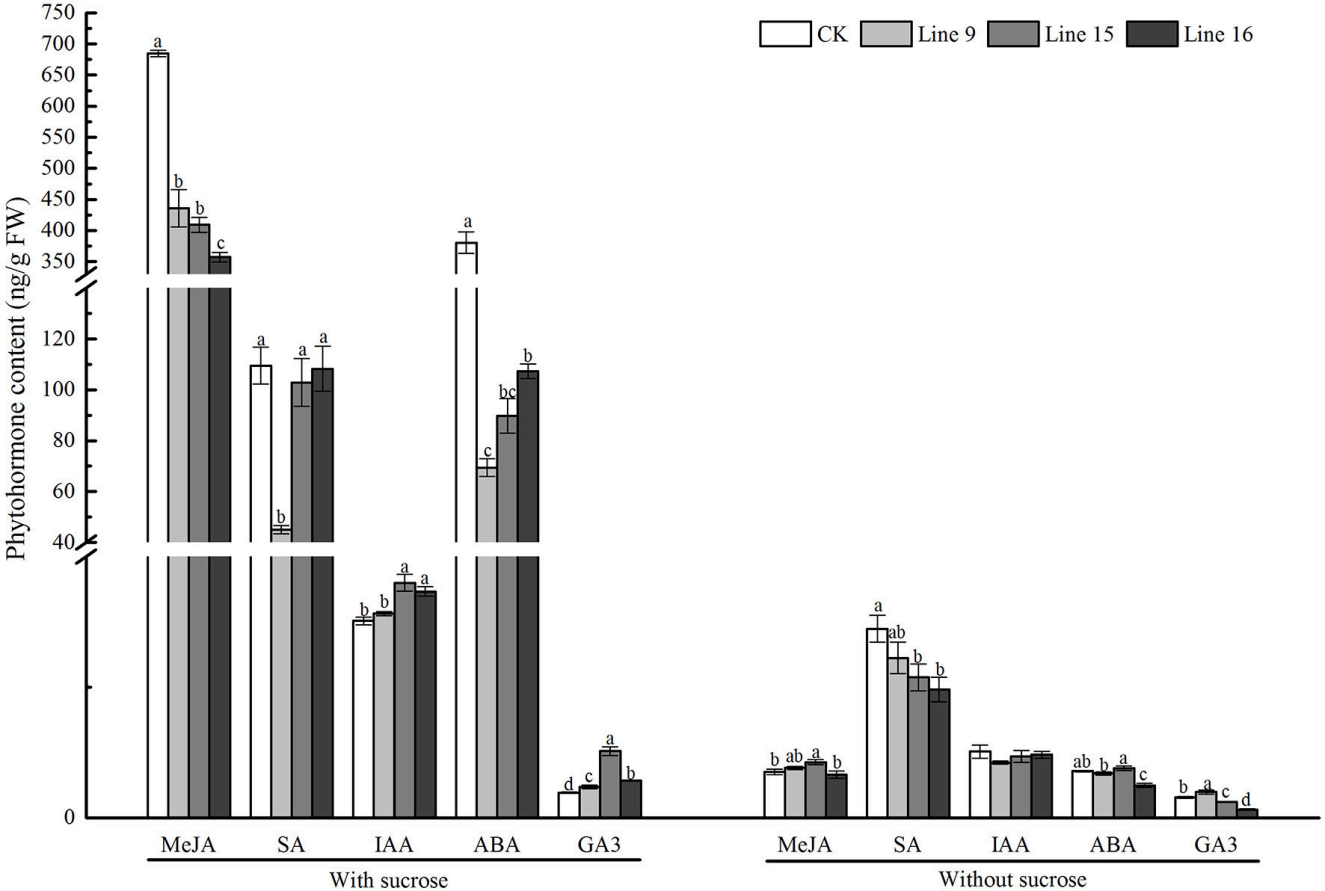
Phytohormone content of VvSUC27-transformed tobacco plants compared with that of CK. The plants were grown *in vitro* for 7 weeks after being plated on modified MS medium containing 30 g·L^−1^ sucrose or without sucrose. Data are expressed as the mean ± *SD* from three independent experiments. Different letters indicate significant differences (*P* < 0.05) differences between transformants (Lines 9, 15, and 16) and CK, as determined by one-way analysis of variance followed by Tukey's test using SPSS statistical software.

The major plant hormones include growth-promoting phytohormones GA, and IAA, as well as stress-mediating phytohormones ABA, JA, MeJA, and SA (Hossain et al., 2011; Trapp et al., 2014; Shahzad et al., 2015). In the present study, compared with CK, the VvSUC27 overexpressing lines planted on sucrose medium showed higher levels of growth-promoting phytohormones (all showed significantly higher GA_3_ levels; two lines showed significantly higher IAA levels) and lower levels of stress-mediating phytohormones (all lines showed significantly higher levels of ABA and MeJA, and one line showed significantly lower levels of SA; Figure 9). Under the control condition, the plants grown on no-sucrose medium showed no significant differences in phytohormone concentration (Figure 9).

### Changes in the expression of *NtSUT1* in *VvSUC27* overexpression lines

**Figure 10:**
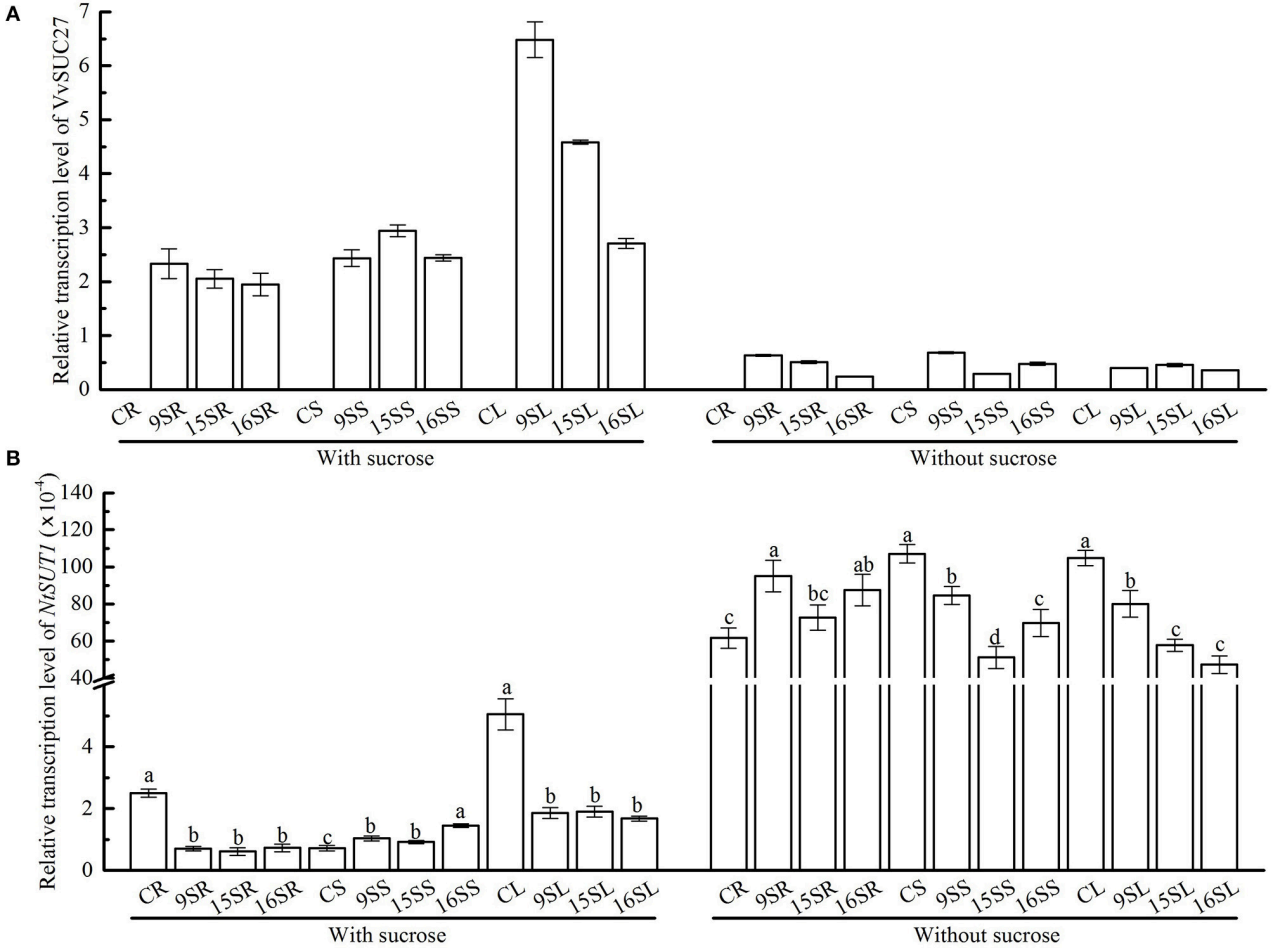
Determination of the relative transcript levels of *VvSUC27* and *NtSUT1*. The plants were grown *in vitro* for 7 weeks after being plated on modified MS medium containing 30 g·L^−1^ sucrose or without sucrose. Transcript ratios of *VvSUC27* **(A)** and *NtSUT1* **(B)** in transformants (Lines 9, 15, and 16) and CK were determined. The roots, stems, and leaves of transformants and CK were obtained. Total RNAs from different tissues were isolated, and transcript levels of the internal control *Nt-EF1*α were used as a standard. Data are expressed as the mean ± *SD* from six independent experiments. Different letters indicate significant differences (*P* < 0.05) differences between transformants (Lines 9, 15, and 16) and CK, as determined by one-way analysis of variance followed by Tukey's test using SPSS statistical software.

In our study, *VvSUC27* cDNA-transformed tobacco plants showed a change in their growth pattern and performance as well as their ability to acquire exogenous sucrose, with an increased sugar content observed in the roots and stems of transformants grown on the sucrose medium. When grown on the MS medium without sucrose, these changes were minimal; therefore, we investigated the expression of *VvSUC27* in transformants grown on MS medium with and without sucrose (Figure 10A). The expression of *VvSUC27* in all parts of transformants was high. Because sucrose is a very important signaling molecule in assimilate partitioning (Chiou and Bush, 1998), and several studies have shown that exogenous sucrose leads to a shift in the expression of SUTs in plants (Weber et al., 1997; Aoki et al., 1999; Matsukura et al., 2000), we investigated the expression of tobacco SUT (*NtSUT1*) in the different plant parts (root, stem, and leaf) of transformants by using real-time PCR (Figure 10B).

Lines 9, 15, and 16 exhibited the same expression patterns for all genes in all organs. RNA isolated from CK was also investigated as a negative control. When grown on the medium containing sucrose, NtSUT1 transcript levels were significantly lower in the roots and leaves of transformants, whereas they were markedly increased in the stems. However, when grown on medium without sucrose, NtSUT1 transcript levels were increased in the roots and decreased in both the stems and leaves of transformants. Furthermore, *NtSUT1* expression was significantly up-regulated in the same organ of the plants in the absence of exogenous sucrose.

### *VvSUC27* overexpression lines showed increased tolerance to multiple abiotic stresses

**Figure 11:**
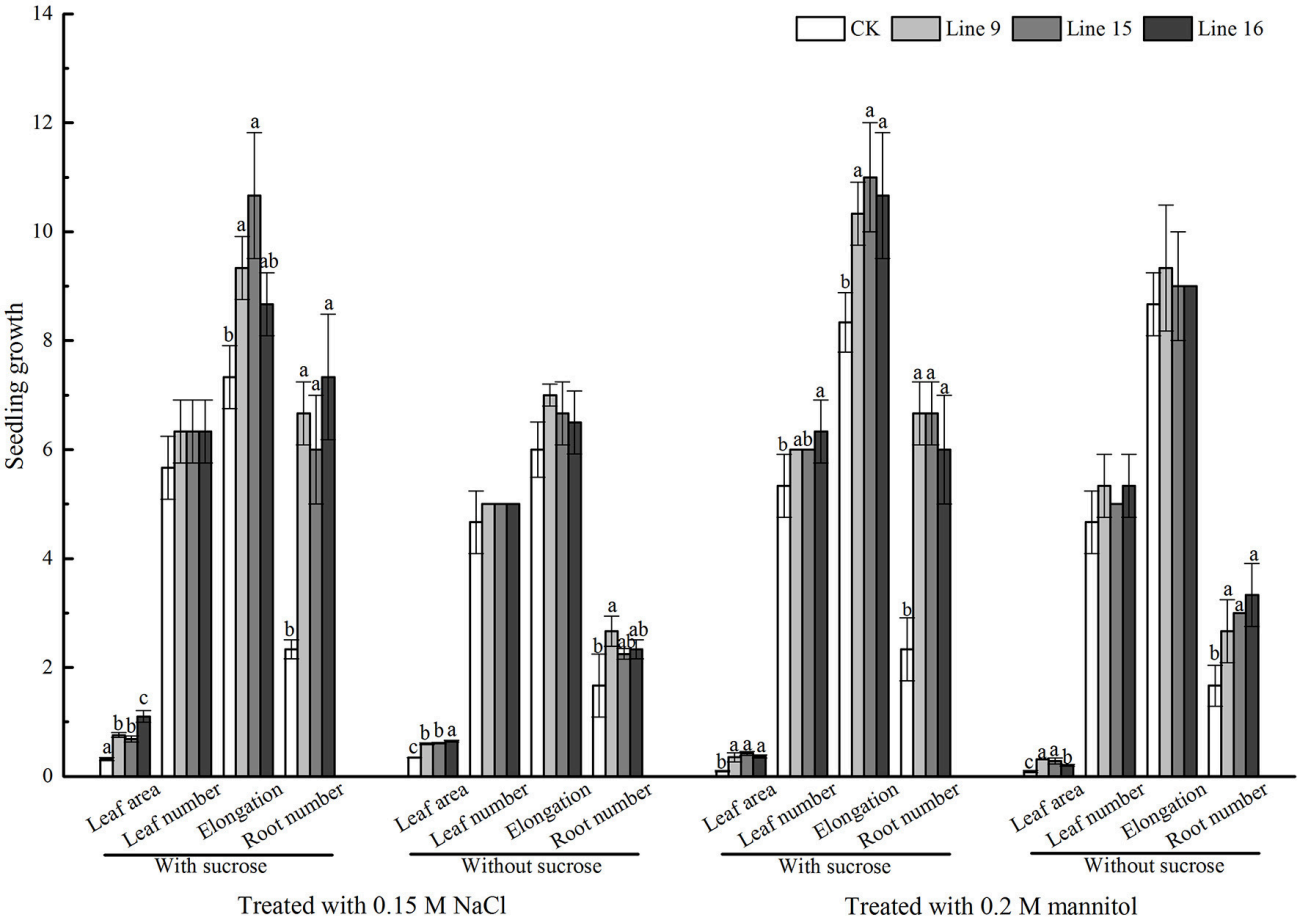
VvSUC27 overexpression lines under abiotic stress. Phenotypic differences in terms of the leaf area, leaf number, elongation, and root number in MS media containing 30 g·L^−1^ sucrose or without sucrose under normal conditions, NaCl, or mannitol from the start of the experiment for 30 d. Different letters indicate significant differences (*P* < 0.05) differences between transformants (Lines 9, 15, and 16) and CK, as determined by one-way analysis of variance followed by Tukey's test using SPSS statistical software.

**Figure 12:**
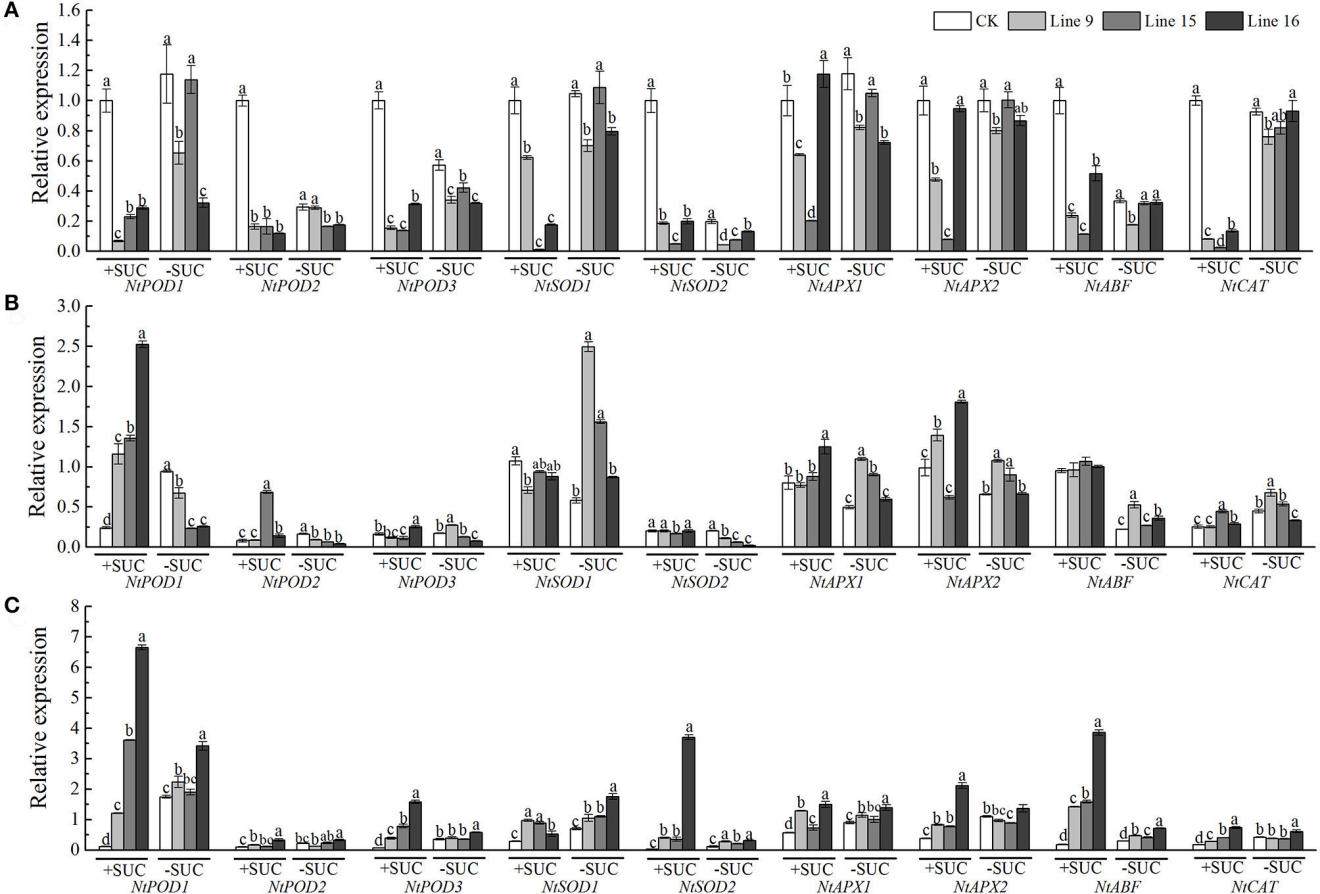
Expression levels of ROS scavengers and ABA-related genes in untransformed (CK) or transgenic tobaccos. Seedling growth in MS media containing 30 g·L^−1^ sucrose or without sucrose under normal conditions **(A)**, NaCl **(B)**, or mannitol **(C)** from the start of the experiment for 30 d. Thirty-day-old seedlings were used to determine the differences in expression between transformants (Lines 9, 15, and 16) and CK. Total RNA from different tissues was isolated and tested for the presence of *Nt-EF1*α transcripts, which served as an internal control. Different letters indicate significant differences (*P* < 0.05) differences between transformants (Lines 9, 15, and 16) and CK, as determined by one-way analysis of variance followed by Tukey's test using SPSS statistical software.

To explore the possible function of *VvSUC27* in providing tolerance to abiotic stress in plants, we planted transformants and CK on medium with and without sucrose with (under salt and mannitol conditions) or without stress (phenotypes had been shown in Figure 5A). The transformants grown on both sucrose and no-sucrose MS medium showed a stronger phenotype to CK under stresses (Figure 11). We further investigated the transcriptional levels of nine reactive oxygen species (ROS) scavengers and ABA-related genes of transformants and CK grown under control or abiotic stress conditions (Figure 12 and Table S2). The genes in most of the transformants were significantly down-regulated under the control condition compared with that in CK (Figure 12A). Most of the genes in most *VvSUC27* overexpression lines were up-regulated after exogenous NaCl treatment (Figure 12B). While under exogenous mannitol treatment, almost all the genes in the transformants were significantly up-regulated (Figure 12C), especially on sucrose MS medium. Then, we compared all factors for each gene measured to find that most genes were down-regulated in CK, while up-regulated in transgenic lines under stress than under the control condition. Most gene regulations presented significant differences, especially on sucrose MS medium (Table S2). The transformants also showed better performance compared to CK under low light and dark conditions when grown on the sucrose MS medium (Figure S2). The transformants had more roots, thicker and longer stems, and more and larger leaves. Moreover, the weaker the light, the longer and stronger were the stems and roots.

## Discussion

Sucrose is the primary product of photosynthetic CO_2_ fixation and is used for the distribution of assimilated carbon within higher plants. Transmembrane transport of sucrose requires the participation of SUTs. Although various studies have focused on SUTs of higher plants, relatively few have been performed on SUTs of *V. vinifera*, especially in terms of their function. In this study, VvSUC27, a SUT located at the plasma membrane, was found to show decreased transcript abundance during berry development and was involved in the promotion of seedling growth, sucrose absorption, sugar, and endogenous phytohormone distribution, and abiotic stress tolerance in the presence of MS medium containing exogenous sucrose.

### Transcript abundance of *VvSUC27* decreases and is negatively correlated with sugar accumulation during berry development

In a previous study, VvSUC27 was described as a LAHC SUT, which might be responsible for phloem loading and sugar retrieval during long-distance transport, with a *K*_*m*_ value for sucrose ranging between 8.0 and 10.5 mM (Zhang et al., 2008; Afoufa-Bastien et al., 2010). Transcript analysis during berry development revealed that four varieties of *V. vinifera* showed relatively high expression levels of VvSUC27 in berries prior to veraison; however, after veraison, its expression in berries decreased remarkably (Davies et al., 1999; Deluc et al., 2007; Afoufa-Bastien et al., 2010; Pastenes et al., 2014; Xu et al., 2015). In this study, we used these four *V. vinifera* varieties to analyze the changes in the expression of *VvSUC27* and their relationship with sugar content during berry development. With an increase in sugar content, the transcript abundance reduced remarkably during berry maturation (Figures 1A–D). Correlation analysis revealed a strong negative correlation (Figure 1B). The onset of ripening involves a shift in phloem unloading from the symplasmic to apoplasmic pathway, and, during that period, a small portion of plasmodesmata and phloem strands are apparently blocked in the ripening stage and during the late mature stage, respectively (Zhang et al., 2006). As an LAHC sink-specific protein, VvSUC27 is responsible for phloem loading and sugar retrieval during long-distance transport (Zhang et al., 2008; Afoufa-Bastien et al., 2010). Therefore, we inferred that the decreasing transcript level of *VvSUC27* might be consistent with the blocked plasmodesmata and phloem strands.

### VvSUC27 plays an important role in improving the absorption of sucrose, determining the rate of seedling growth, and promoting the growth and development of transgenic tobacco in the presence of exogenous sucrose

As noted, VvSUC27 exhibited sucrose transport activity across the plasma membrane when expressed in yeast (Zhang et al., 2008). Sucrose-[^14^C] uptake (Figure 2) and subcellular localization (Figure 3) were successfully monitored in the present study. The total rate of sucrose uptake in transgenic plants containing VvSUC27 increased, indicating their enhanced ability for sucrose loading, which provides further evidence that VvSUC27 is a plasma membrane sucrose transporter.

Sucrose can induce meristem quiescence as observed in the arrested development of seedlings germinated on high concentrations of sucrose (Lastdrager et al., 2014). Investigations on the effect of different sucrose concentrations on the growth of transgenic and CK seedlings showed that the higher the sucrose concentration, the stronger the rate of tobacco seedling growth inhibition, and the transformed plants were more sensitive to high sucrose concentrations (Figure 4). The inhibitory effects on early seedling development might be owing to osmotic stress, a hexokinase-independent mechanism, or ABA (Gibson, 2005). Interestingly, given sufficient time, the majority of seeds might germinate on even very high concentrations of sugars, which might be because of ABA metabolism (Laby et al., 2000; Cho and Yoo, 2011).

Many studies have suggested that reduced expression of sucrose transporters has deleterious effects on plant growth and development; however, improvement in plant performance in response to enhanced expression of the SUT has not yet been reported. Despite the higher protein content of HOSUT grains, no statistically significant effect was noted on wheat grains (Weichert et al., 2010). In our study, compared with plants in normal conditions, although the small volume of the vessel may inhibit the plant growth, resulting in smaller leaves, shorter stems, and weaker roots, there were still prominent phenotypic differences between transformants and CK (Figure 5), with VvSUC27-transformed plants exhibiting more rapid and stronger development. These enhanced plant morphologies resulted in a higher dry weight of the separate parts (leaves, stems, and roots) and a lower shoot/root ratio of the transgenic tobacco plants compared with that in CK (Figure 6). The decreased dry weight/seedling ratio of leaves and increased ratio of stems and roots in the transformants indicated that the main energy pathway switched from autotrophy to heterotrophy (Figure 6B). Transformants were also geminated under the same conditions used previously, except MS medium with no sucrose. Thus, the development of transformants was visually indistinct from that of CK (Figures 5, 6). We elucidated that sucrose uptake was the most important factor in the development of the enhanced phenotype.

### VvSUC27 alters the storage of carbon and phytohormones as well as the expression of endogenous genes in transgenic tobacco in the presence of exogenous sucrose

Sucrose is the main transported form of assimilates; however, it also regulates various processes such as carbon storage. Carbohydrate accumulation in leaves leads to the down-regulation of photosynthesis (Jang and Sheen, 1994). In our study, we planted transformants on sucrose-containing medium and found that the sieve elements were larger and more developed in the roots of transformants than in CK (Figure 7A), indicating the strong absorption and nutrient transport ability of the plants. The sucrose concentration did not increase in the leaves of transformants (Figure 8). However, the leaves changed their color to yellow, and chloroplasts in leaf cells were smaller and decreased in number compared with those in CK (Figure 7B), indicating that inactivate metabolism in the chloroplasts was the most likely reason for the observed chlorosis of the transformed leaves. Degeneration of chloroplasts indicated that the leaves of the transformants had impaired carbon fixation, resulting in reduced levels of carbohydrate metabolites fixed from the atmosphere. Furthermore, the expression of the photosynthetic gene *lhcb* was significantly inhibited in the leaves (Figure 7C). The most important factor affecting photosynthetic capacity was the nitrogen absorption ability of roots, rather than the total non-structural carbohydrates (Sugiura et al., 2015), indicating that the high sucrose concentration might account for the high C/N ratio of the whole plant and influence the nitrogen supply in roots, resulting in the inhibition of photosynthetic gene expression and changes in the photosynthetic capacity of the leaves. Our results showed that, when the roots of a transformed plant uptake sufficient carbohydrate, the plants restrain the function of the leaves to fix carbon dioxide from the atmosphere to maintain an optimal growth status. The chlorosis observed in mature leaves might explain this result because plants tend to maintain steady homeostasis. In our study, *VvSUC27* was under the control of the 35S promoter; therefore, it might have been expressed in all tissues of the transformants. It was found to act as a SUT in the roots, with more than 2-fold of sugar from outside MS sucrose medium in the excess of CK. The sugar concentration decreased in the stems and leaves, indicating that plants can regulate their sugar content within an appropriate range for optimal growth. When grown on medium without sucrose, the sugar content of the plants was considerably lower than that when grown on the sucrose medium; further, no consistent differences were found between the transformants and CK. Furthermore, when sucrose in MS media was nearly exhausted after the plants had been grown over 8–9 weeks, the color of the leaves from transformants changed to green (data not shown). However, the transformants planted on the no-sucrose MS medium showed little difference in their sugar content (Figure 8) and in the expression of *lhcbs* (Figure 7C), compared to those grown on MS medium containing sucrose.

JA, MeJA, ABA, and SA are the key signaling compounds involved in the responses of plants to biotic and abiotic stresses, as well as in development (Wasternack, 2014; Shahzad et al., 2015). Enhanced plant growth characteristics have been attributed to increased IAA and a marked decrease in ABA (Shahzad et al., 2015). Stress-mediating hormones (MeJA, ABA, and SA) are induced in response to mechanical wounding, insect herbivory, and other abiotic and biotic stresses (Santner and Estelle, 2009). However, this beneficial effect is often offset by slowed growth (Shahzad et al., 2015). In our study, the concentration of most of the stress-mediating hormones (MeJA, ABA, and SA) decreased, whereas that of the growth-promoting hormones increased (GA_3_ and IAA) in the transformants grown on the sucrose MS medium (Figure 9). Therefore, we inferred that VvSUC27 promoted the growth of the transformants, leading to an increase in the levels of growth-promoting hormones, which antagonized the effects of the stress-mediating hormones. Furthermore, this rapid growth inhibited the synthesis of stress-mediating hormones. The phytohormones of transformants planted on the no-sucrose MS medium were indistinct from those of CK.

Sugars can affect the ribosomal protein paralog composition of ribosomes so that they act as regulatory signals that could affect gene translational activity (Hummel et al., 2012). The changes in sugar levels might affect the activity and expression of the sugar transporter. In citrus (Yao Li et al., 2003), the expression of sucrose transporters was suppressed at high concentrations of sugar. However, in rice embryo, *OsSUT1* expression was enhanced by the increased levels of endogenous sugar, as well as light exposure (Matsukura et al., 2000). In the present study, all the transformants had higher expression of *VvSUC27* (Figure 10A). In the sucrose medium, the expression of *NtSUT1* was significantly suppressed in the roots of transformants, but markedly enhanced in the stems (Figure 10B). However, in the leaves, the expression of NtSUT1 decreased inconspicuously (Figure 10B), probably because the sucrose concentration in the leaves of transformants did not vary as much as that in CK. The transgenic tobacco plants might have attempted to restrict the amount of sucrose taken up via the roots and restricted the expression of *NtSUT1*. Further, abundant sucrose needs to be transported from the root to the other organs, and the expression of *NtSUT1* in the stem was thus enhanced. Thus, NtSUT1 is essential for sucrose export, even when sugar biosynthesis was reduced or soluble sugars accumulated (Burkle et al., 1998). In this study, we compared the expression level of *NtSUT1* in medium with and without sucrose and found that NtSUT1 was highly upregulated in the presence of low levels of exogenous sucrose (Figure 10B), which was consistent with the findings for AtSUC9 in *Arabidopsis* (Jia et al., 2015). When grown in the no-sucrose medium, the expression of *NtSUT1* in the transformants was slightly reduced in the roots; however, it was increased in the stems and leaves (Figure 10B). Compared with that in CK, the transformants might have attempted to increase the uptake of sucrose via the roots in the no-sucrose condition; therefore, it released the expression of its *NtSUT1*. Since, high levels of sucrose were not absorbed, no transport from the root to the other organs was needed. Thus, in the stem, there was no need for enhanced expression of *NtSUT1*.

### VvSUC27 plays an important role under multiple abiotic stresses *In vitro*

Source–sink sucrose distribution is known to be beneficial for plant growth and stress tolerance (Roitsch, 1999). Thus far, results on the role of sucrose transporters at the whole-plant level under abiotic stress conditions have revealed a sucrose imbalance in the sink and source, and hypersensitivity in response to abiotic stress treatments when they were induced by defective SUT function, including AtSUC2, AtSUC4, AtSUC9, and PtaSUT4 (Frost et al., 2012; Gong et al., 2015; Jia et al., 2015). This suggests that sucrose transporters might be involved in the redistribution of sucrose, which is useful for plants to resist abiotic stress. To confirm the positive role of VvSUC27 in resistance to abiotic stress, we planted transformants and CK on medium with and without sucrose under stress conditions. The transformants grown on both sucrose and no-sucrose MS medium showed a stronger phenotype to CK (Figure 11). ABF acts as an ABA-responsive signaling protein to activate antioxidant defense mechanisms in plants (Ji et al., 2013). ROS levels increase immediately when chelated under abiotic stress, and their scavenging is thought to contribute toward enhancing abiotic stress tolerance in plants (Huang et al., 2010; Yoshida et al., 2015). Therefore, we investigated the transcriptional levels of nine ROS scavengers and ABA-related genes of transformants and CK grown under control or abiotic stress conditions (Figure 12 and Table S2). Most of the genes in the VvSUC27 overexpression lines were significantly up-regulated under stress conditions, especially on sucrose MS medium, and markedly changes could indeed be found in the sucrose content of different parts (Figure 8B) and in the transcriptional levels of nine ROS scavengers and ABA-related genes (Figure 12A), compared with CK in the normal condition. Thus, we inferred that the enhanced stress resistance of VvSUC27 overexpressing lines might be involved in plant abiotic stress tolerance largely dependent on regulating the sucrose content in the source and sink, which might be related to the ABA signaling pathway and to ROS scavenging enzymes.

## Author contributions

YC, JL, and YLZ designed the research. YC, WT, YYZ, JY, and ZX performed the research. YC and YLZ drafted the article.

### Conflict of interest statement

The authors declare that the research was conducted in the absence of any commercial or financial relationships that could be construed as a potential conflict of interest.
