# Corrigendum: Overexpression of a Grapevine Sucrose Transporter (VvSUC27) in Tobacco Improves Plant Growth Rate in the Presence of Sucrose *In vitro*

**DOI:** 10.3389/fpls.2017.01817

**Published:** 2017-10-16

**Authors:** Yumeng Cai, Wenrui Tu, Yunyun Zu, Jing Yan, Zimo Xu, Jiang Lu, Yali Zhang

**Affiliations:** ^1^Beijing Advanced Innovation Center for Food Nutrition and Human Health, College of Food Science and Nutritional Engineering, China Agricultural University, Beijing, China; ^2^Center for Viticulture and Enology, School of Agriculture and Biology, Shanghai Jiao Tong University, Shanghai, China

**Keywords:** grapevine, VvSUC27, growth, abiotic stresses, sucrose

An author name was incorrectly spelled as [Yan Jing]. The correct spelling is [Jing Yan]. The authors apologize for this error and state that this does not change the scientific conclusions of the article in any way.

## Conflict of interest statement

The authors declare that the research was conducted in the absence of any commercial or financial relationships that could be construed as a potential conflict of interest.

