# Corrigendum: Overexpression of a Grapevine Sucrose Transporter (VvSUC27) in Tobacco Improves Plant Growth Rate in the Presence of Sucrose *In vitro*

**DOI:** 10.3389/fpls.2018.00036

**Published:** 2018-02-05

**Authors:** Yumeng Cai, Wenrui Tu, Yunyun Zu, Jing Yan, Zimo Xu, Jiang Lu, Yali Zhang

**Affiliations:** ^1^Beijing Advanced Innovation Center for Food Nutrition and Human Health, College of Food Science and Nutritional Engineering, China Agricultural University, Beijing, China; ^2^Center for Viticulture and Enology, School of Agriculture and Biology, Shanghai Jiao Tong University, Shanghai, China

**Keywords:** grapevine, VvSUC27, growth, abiotic stresses, sucrose

In the original article, there was a mistake in the legend for **Figure 7** as published. We stated “Karyons (deep red dots) were stained with DAPI staining solution, whereas leaf chloroplasts (green dots) were stained with hematoxylin and eosin (HE)” the method was written wrongly. The correct legend appears below.

**Figure 7**. Cross-section of the root and leaf and expression of chlorophyll-related genes in VvSUC27 transformants and CK. Organic slice analysis of root (A) and leaf (B). Organic slices were stained by Safranin O/Fast Green staining (SO/FG). Karyons, sieve element, and chloroplasts are indicated by red, blue, and white arrowheads, respectively. Transcript ratios of light harvesting chlorophyll a/b-binding protein (*lhcb*) in transformants (Lines 9, 15, and 16) compared with that in CK **(C)**. The plants were grown *in vitro* for 7 weeks after being plated on MS medium containing 30 g·L^−1^ sucrose. Data are expressed as the mean ± SD from six independent experiments. Different letters indicate significant differences (*P* < 0.05) differences between transformants (Lines 9, 15, and 16) and CK, as determined by one-way analysis of variance followed by Tukey's test using SPSS statistical software.

In the original article, there was an error. The first sentence in the **RESULTS**, ***VvSUC27*** **overexpression lines showed increased tolerance to multiple abiotic stresses, describing Figure 5A, but written wrongly as Figure 3A**. A correction has been made to **RESULTS**, ***VvSUC27* overexpression lines showed increased tolerance to multiple abiotic stresses, Paragraph 1:**

To explore the possible function of *VvSUC27* in providing tolerance to abiotic stress in plants, we planted transformants and CK on medium with and without sucrose with (under salt and mannitol conditions) or without stress (phenotypes had been shown in **Figure 5A**). The transformants grown on both sucrose and no-sucrose MS medium showed a stronger phenotype to CK under stresses (Figure 11). We further investigated the transcriptional levels of nine reactive oxygen species (ROS) scavengers and ABA-related genes of transformants and CK grown under control or abiotic stress conditions (Figure 12 &Table S2). The genes in most of the transformants were significantly down-regulated under the control condition compared with that in CK (Figure 12A). Most of the genes in most *VvSUC27* overexpression lines were up-regulated after exogenous NaCl treatment (Figure 12B). While under exogenous mannitol treatment, almost all the genes in the transformants were significantly up-regulated (Figure 12C), especially on sucrose MS medium. Then we compared all factors for each gene measured to find that most genes were down-regulated in CK, while up-regulated in transgenic lines under stress than under the control condition. Most gene regulations presented significant differences, especially on sucrose MS medium (Table S2). The transformants also showed better performance compared to CK under low light and dark conditions when grown on the sucrose MS medium (Supplementary Figure S2). The transformants had more roots, thicker and longer stems, and more and larger leaves. Moreover, the weaker the light, the longer and stronger were the stems and roots.

The original article has been updated, including the correction mentioned in another corrigendum article doi: 10.3389/fpls.2017.01817.

The authors apologize for these errors and state that this does not change the scientific conclusions of the article in any way.

## Conflict of interest statement

The authors declare that the research was conducted in the absence of any commercial or financial relationships that could be construed as a potential conflict of interest.

