# Supplementary material for: Overexpression of a Grapevine Sucrose Transporter (VvSUC27) in Tobacco Improves Plant Growth Rate in the Presence of Sucrose In vitro
Source: Front Plant Sci. 2017 Jun 20;8:1069. doi: 10.3389/fpls.2017.01069 (PMC5476780; doi:10.3389/fpls.2017.01069)
Supplement: Supplementary file 1 [file Table1.PDF]

Table S1. Primers used in the study.

**Target gene**	**Sequence 5′→ 3′**	
VvSUC27 F1	CCGCTCGAGATGGAGTTAGCCAAGCCTTCT	Used for pUC19-35S
VvSUC27 R1	CCGTTCGAATTACTTGTACAGCTCGTCCATGC	
VvSUC27 F2	TGCTCTAGAATGGAGTTAGCCAAGCCTTCTTC	Used for pBI121
VvSUC27 R2	TCCCCCCGGGTAGACGACGGCTGAGTCCT	
NtSUT1+	AAAAGGGTCCAAACCTCGA	Used for RT-PCR analysis
NtSUT1-	TCGTCGATTTCGTGTTCG	
VvSUC27+	CTGACAATCATCGCATTCGCC	
VvSUC27-	GAAGAGAAGGAAAGGGAACCA	
lhcb1+	ATGGGCTTGATTGAAGGATA	
lhcb1-	ATCGTCCGTTCTTGATTTCC	
lhcb13+	AGCGAAGGAGGACTTGACTA	
lhcb13-	AAAAGTTGTCGGGTCATCGG	
NT- EF1α+	GATCCTTAACCGCAACATTCTT	
NT- EF1α-	CCTCAAGCCTGGTATGGTTGT	
NtPOD1+	GTTTCTGAATACAGTAACAGCCCTCG	
NtPOD1-	GACACTGCCACAGACTTTTCTTATGA	
NtPOD2+	TTTGTTCAGGGTTGTGACGGTTCT-3’	
NtPOD2-	5’-TTCTTCCAGTAGGCACTCCCCA-3’	
NtPOD3+	5’-GGGAATGAAAGATTTGGTTGCTC-3’	
NtPOD3-	5’-TGCTATCTCTGTTTGGTTTGGGG-3’	
NtSOD1+	5’-GAATCAATGAAGCCCAACGGA-3’	
NtSOD1-	5’-TCTATGGTGAGGAGCGGTGTGTA-3’	
NtSOD2+	5’-ACAAGAAACTCTGCACTTGTGATGG-3’	
NtSOD2-	5’-GAGCAACTCTGTAAATAGGAACCAAG-3’	
NtAPX1+	5’- CCACCAAGGGTTCTGACCATCT -3’	
NtAPX1-	5’- CCAATGGGCGGAAGACAGG -3’	
NtAPX2+	5’- GACCTGATGTTCCCTTTCACCCTG -3’	
NtAPX2-	5’- CAGATAGACCCATTTGCTTCACA -3’	
NtABF+	5’- TTCAAGGCAGTGTTATGCAGGGT -3’	
NtABF-	5’- CGTCTTCTTTCCACAACTTTTTCCA -3’	
NtCAT+	5’- CTCGCTTTCTGCCCTTCTATTGT -3’	
NtCAT-	5’- GCACTTTGGAGCATTAGCAGGA -3’	

+, sense sequence; -, anti-sense sequence.
