# Supplementary material for: Overexpression of a Grapevine Sucrose Transporter (VvSUC27) in Tobacco Improves Plant Growth Rate in the Presence of Sucrose In vitro
Source: Front Plant Sci. 2017 Jun 20;8:1069. doi: 10.3389/fpls.2017.01069 (PMC5476780; doi:10.3389/fpls.2017.01069)
Supplement: Supplementary file 2 [file Table2.PDF]

Table S2. The values of relative expression levels of ROS scavengers and ABA-related genes in untransformed (CK) or transgenic tobaccos. Seedling growth in MS media containing 30 g·L^{-1} sucrose or without sucrose under normal conditions, NaCl, or mannitol from the start of the experiment for 30 d. Thirty-day-old seedlings were used to determine the differences in expression between transformants (Lines 9, 15, and 16) and CK. Total RNA from different tissues was isolated and tested for the presence of *Nt-EF1α* transcripts, which served as an internal control.

		Under normal conditions				Under 0.15 M NaCl treatment				Under 0.2 M mannitol treatment			
		CK	Line 9	Line 15	Line 16	CK	Line 9	Line 15	Line 16	CK	Line 9	Line 15	Line 16
NtPOD1	+	1.00 ± 0.08^{gh}	0.07 ± 0.03^{j}	0.23 ± 0.01^{j}	0.29 ± 0.01^{ij}	0.24 ± 0.01^{j}	1.16 ± 0.12^{fgh}	1.36 ± 0.03^{efg}	2.53 ± 0.04^{c}	0.12 ± 0.01^{j}	1.21 ± 0.01^{fgh}	3.62 ± 0.25^{b}	6.66 ± 0.54^{a}
	-	1.18 ± 0.19^{fgh}	0.65 ± 0.07^{hij}	1.14 ± 0.09^{fgh}	0.32 ± 0.03^{ij}	0.94 ± 0.01^{ghi}	0.68 ± 0.07^{hij}	0.23 ± 0.01^{j}	0.26 ± 0.01^{j}	1.75 ± 0.12^{def}	2.24 ± 0.18^{cd}	1.91 ± 0.11^{ced}	3.42 ± 0.14^{b}
NtPOD2	+	1.00 ± 0.04^{a}	0.16 ± 0.02^{efg}	0.16 ± 0.04^{efg}	0.12 ± 0.01^{fgh}	0.08 ± 0.02^{gh}	0.09 ± 0.01^{fgh}	0.68 ± 0.02^{b}	0.14 ± 0.02^{fg}	0.11 ± 0.01^{fgh}	0.17 ± 0.01^{ef}	0.12 ± 0.01^{fgh}	0.33 ± 0.04^{c}
	-	0.29 ± 0.02^{cd}	0.28 ± 0.01^{cd}	0.16 ± 0.01^{efg}	0.17 ± 0.02^{ef}	0.16 ± 0.02^{efg}	0.09 ± 0.03^{fgh}	0.07 ± 0.03^{h}	0.05 ± 0.01^{h}	0.22 ± 0.05^{de}	0.13 ± 0.04^{fgh}	0.24 ± 0.03^{de}	0.33 ± 0.03^{c}
NtPOD3	+	1.00 ± 0.05^{b}	0.16 ± 0.02^{hij}	0.14 ± 0.03^{ij}	0.31 ± 0.01^{efg}	0.16 ± 0.02^{hij}	0.12 ± 0.01^{j}	0.11 ± 0.02^{j}	0.25 ± 0.02^{ghi}	0.07 ± 0.01^{j}	0.39 ± 0.03^{ef}	0.79 ± 0.06^{c}	1.58 ± 0.16^{a}
	-	0.57 ± 0.03^{d}	0.34 ± 0.02^{efg}	0.42 ± 0.01^{e}	0.32 ± 0.02^{efg}	0.17 ± 0.01^{hij}	0.28 ± 0.02^{fgh}	0.13 ± 0.01^{ij}	0.07 ± 0.02^{j}	0.36 ± 0.02^{efg}	0.40 ± 0.01^{ef}	0.37 ± 0.01^{efg}	0.59 ± 0.02^{d}
NtSOD1	+	1.00 ± 0.09^{cde}	0.62 ± 0.01^{fgh}	0.01 ± 0.00^{j}	0.31 ± 0.01^{ij}	1.08 ± 0.25^{cd}	0.71 ± 0.04^{efgh}	0.94 ± 0.02^{cde}	0.88 ± 0.05^{cdefg}	0.29 ± 0.01^{ij}	0.97 ± 0.03^{cde}	0.89 ± 0.04^{cdef}	0.54 ± 0.11^{hi}
	-	1.05 ± 0.02^{cd}	0.70 ± 0.04^{efgh}	1.09 ± 0.11^{cd}	0.80 ± 0.03^{defgh}	0.58 ± 0.02^{ghi}	2.50 ± 0.26^{a}	1.56 ± 0.12^{b}	0.87 ± 0.01^{cdefg}	0.70 ± 0.04^{efgh}	1.06 ± 0.12^{cd}	1.11 ± 0.07^{c}	1.76 ± 0.10^{b}
NtSOD2	+	1.00 ± 0.08^{b}	0.19 ± 0.01^{defgh}	0.05 ± 0.00^{gh}	0.20 ± 0.02^{defgh}	0.20 ± 0.01^{defgh}	0.21 ± 0.01^{defgh}	0.16 ± 0.00^{defgh}	0.22 ± 0.02^{cdefg}	0.04 ± 0.00^{gh}	0.41 ± 0.01^{c}	0.35 ± 0.00^{cd}	3.71 ± 0.28^{a}
	-	0.20 ± 0.01^{defgh}	0.04 ± 0.00^{gh}	0.08 ± 0.01^{gh}	0.13 ± 0.02^{efgh}	0.20 ± 0.03^{defgh}	0.11 ± 0.01^{fgh}	0.06 ± 0.01^{gh}	0.02 ± 0.00^{h}	0.12 ± 0.03^{fgh}	0.28 ± 0.02^{cdef}	0.21 ± 0.01^{defgh}	0.32 ± 0.01^{cde}
NtAPX1	+	1.00 ± 0.10^{efgh}	0.64 ± 0.01^{jkl}	0.20 ± 0.02^{m}	1.18 ± 0.09^{bcde}	0.80 ± 0.07^{hijk}	0.77 ± 0.12^{ijk}	0.87 ± 0.02^{ghi}	1.25 ± 0.09^{bcd}	0.58 ± 0.01^{kl}	1.29 ± 0.01^{abc}	0.74 ± 0.09^{ijk}	1.50 ± 0.10^{a}
	-	1.17 ± 0.11^{cde}	0.82 ± 0.02^{hij}	1.05 ± 0.02^{defg}	0.72 ± 0.01^{ijkl}	0.50 ± 0.01^{l}	1.10 ± 0.12^{cdef}	0.91 ± 0.02^{fghi}	0.60 ± 0.02^{jkl}	0.90 ± 0.04^{fghi}	1.15 ± 0.07^{cde}	1.00 ± 0.10^{efgh}	1.39 ± 0.09^{ab}
NtAPX2	+	1.00 ± 0.10^{de}	0.48 ± 0.01^{hi}	0.08 ± 0.00^{j}	0.95 ± 0.02^{def}	0.99 ± 0.10^{de}	1.40 ± 0.09^{c}	0.72 ± 0.06^{fgh}	1.81 ± 0.17^{b}	0.39 ± 0.02^{i}	0.83 ± 0.07^{efg}	0.77 ± 0.04^{efg}	2.12 ± 0.15^{a}
	-	1.00 ± 0.08^{de}	0.80 ± 0.02^{efg}	1.00 ± 0.05^{de}	0.87 ± 0.03^{defg}	0.66 ± 0.01^{gh}	1.08 ± 0.09^{d}	0.95 ± 0.13^{def}	0.67 ± 0.01^{gh}	1.10 ± 0.03^{d}	0.97 ± 0.05^{de}	0.89 ± 0.07^{defg}	1.37 ± 0.12^{c}
NtABF	+	1.00 ± 0.10^{ab}	0.24 ± 0.02^{jl}	0.11 ± 0.00^{m}	0.52 ± 0.05^{de}	0.95 ± 0.03^{b}	0.97 ± 0.09^{ab}	1.07 ± 0.05^{a}	1.01 ± 0.02^{ab}	0.18 ± 0.01^{lm}	0.29 ± 0.02^{ijl}	0.41 ± 0.01^{efgh}	0.74 ± 0.03^{c}
	-	0.33 ± 0.01^{ghij}	0.18 ± 0.01^{lm}	0.32 ± 0.02^{ghij}	0.32 ± 0.01^{ghij}	0.22 ± 0.01^{jlm}	0.53 ± 0.04^{d}	0.28 ± 0.01^{ijl}	0.36 ± 0.03^{fghi}	0.30 ± 0.01^{hij}	0.47 ± 0.01^{def}	0.42 ± 0.02^{defg}	0.72 ± 0.02^{c}
NtCAT	+	1.00 ± 0.03^{a}	0.08 ± 0.00^{mn}	0.02 ± 0.00^{n}	0.14 ± 0.01^{lm}	0.25 ± 0.02^{jk}	0.25 ± 0.01^{jk}	0.44 ± 0.02^{g}	0.30 ± 0.01^{ij}	0.18 ± 0.01^{kl}	0.29 ± 0.02^{ij}	0.40 ± 0.02^{gh}	0.74 ± 0.03^{bc}
	-	0.93 ± 0.02^{a}	0.76 ± 0.05^{bc}	0.82 ± 0.04^{b}	0.93 ± 0.07^{a}	0.45 ± 0.02^{fg}	0.68 ± 0.04^{cd}	0.54 ± 0.02^{ef}	0.34 ± 0.05^{hij}	0.43 ± 0.01^{gh}	0.38 ± 0.04^{ghi}	0.37 ± 0.03^{ghi}	0.62 ± 0.05^{de}

Use the relative quantification and set the expression level of each gene in CK on MS medium containing 30 g·L^{-1} sucrose as 1. Data are expressed as the mean ± S.D. from six independent experiments. Different letters indicate significant differences ($P<0.05$) within one same gene, as determined by one-way analysis of variance followed by Tukey's test using SPSS statistical software. +, MS medium containing 30 g·L^{-1} sucrose. −, MS medium containing no sucrose.
