# Supplementary material for: Overexpression of a Grapevine Sucrose Transporter (VvSUC27) in Tobacco Improves Plant Growth Rate in the Presence of Sucrose In vitro
Source: Front Plant Sci. 2017 Jun 20;8:1069. doi: 10.3389/fpls.2017.01069 (PMC5476780; doi:10.3389/fpls.2017.01069)
Supplement: Supplementary file 3 [file Image1.PDF]

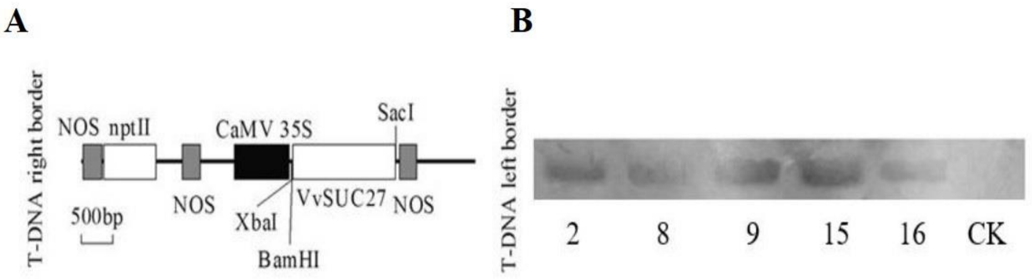

Figure S1. Confirmation of the VvSUC27 expression vector and transgenic tobacco production. Chimeric gene for the expression of VvSUC27 in tobacco (*N. tabacum*) plants **(A)**. Southern blot analysis of transgene expression. DNA was extracted from source leaves of transgenic (2, 8, 9, 15, 16) and control (CK) tobaccoplants **(B)**. The filter was hybridized with a 500-bp fragment encoding the middle part of VvSUC27.
