# Supplementary material for: Overexpression of a Grapevine Sucrose Transporter (VvSUC27) in Tobacco Improves Plant Growth Rate in the Presence of Sucrose In vitro
Source: Front Plant Sci. 2017 Jun 20;8:1069. doi: 10.3389/fpls.2017.01069 (PMC5476780; doi:10.3389/fpls.2017.01069)
Supplement: Supplementary file 4 [file Image2.PDF]

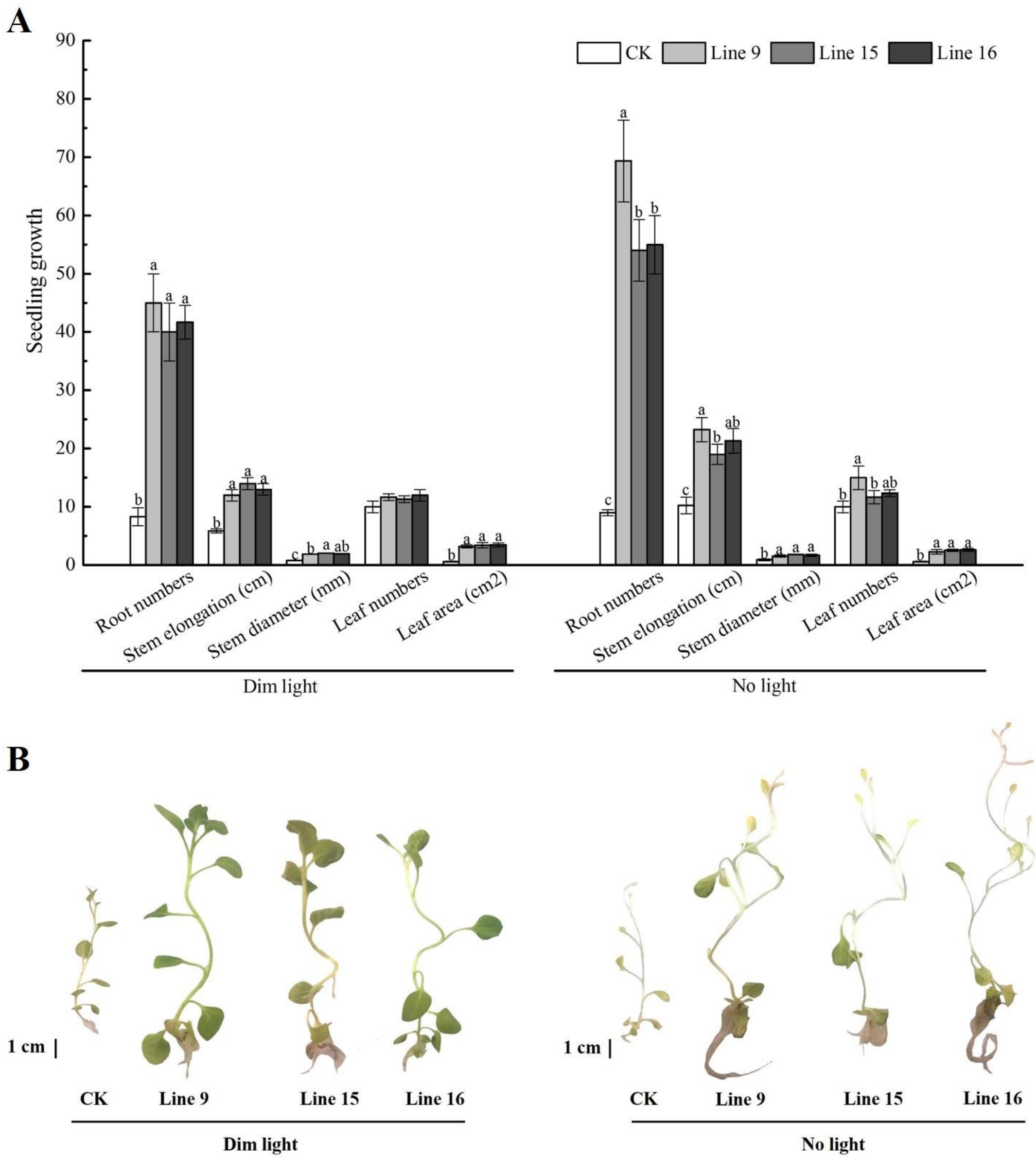

Figure S2 Phenotypes of VvSUC27-transformed tobacco plants under low light or no light conditions. Seedlings were grown in MS media containing 30 g·L^{-1} sucrose for 30 d and then placed under low light or no light conditions for a further 30 d. The seedlings were used to determine differences in root number, stem elongation, stem diameter, leaf number, and leaf area between transformants (Lines9,15, and 16) and CK. Different letters indicate significant differences ($P < 0.05$) differences between transformants (Lines 9, 15, and 16) and CK, as determined by one-way analysis of variance followed by Tukey's test using SPSS statistical software (**A**). The phenotypic development of T2transformants compared with CK following low light or no light treatment **(B)**.
